# Molecular mapping of interstitial lung disease reveals a phenotypically distinct senescent basal epithelial cell population

**DOI:** 10.1172/jci.insight.143626

**Published:** 2021-04-22

**Authors:** Daryle J. DePianto, Jason A. Vander Heiden, Katrina B. Morshead, Kai-Hui Sun, Zora Modrusan, Grace Teng, Paul J. Wolters, Joseph R. Arron

**Affiliations:** 1 Department of Immunology Discovery,; 2 Department of OMNI Bioinformatics, and; 3 Department of Molecular Biology, Genentech Inc., San Francisco, California, USA.; 4 Division of Pulmonary, Critical Care, Allergy and Sleep Medicine, Department of Medicine, University of California, San Francisco, California, USA.

**Keywords:** Aging, Pulmonology, Cellular senescence, Fibrosis

## Abstract

Compromised regenerative capacity of lung epithelial cells can lead to cellular senescence, which may precipitate fibrosis. While increased markers of senescence have been reported in idiopathic pulmonary fibrosis (IPF), the origin and identity of these senescent cells remain unclear, and tools to characterize context-specific cellular senescence in human lung are lacking. We observed that the senescent marker p16 is predominantly localized to bronchiolized epithelial structures in scarred regions of IPF and systemic sclerosis–associated interstitial lung disease (SSc-ILD) lung tissue, overlapping with the basal epithelial markers Keratin 5 and Keratin 17. Using in vitro models, we derived transcriptional signatures of senescence programming specific to different types of lung epithelial cells and interrogated these signatures in a single-cell RNA-Seq data set derived from control, IPF, and SSc-ILD lung tissue. We identified a population of basal epithelial cells defined by, and enriched for, markers of cellular senescence and identified candidate markers specific to senescent basal epithelial cells in ILD that can enable future functional studies. Notably, gene expression of these cells significantly overlaps with terminally differentiating cells in stratified epithelia, where it is driven by p53 activation as part of the senescence program.

## Introduction

Idiopathic pulmonary fibrosis (IPF) is a chronic progressive fibrotic lung disease wherein the distal lung tissue is mired in a perpetual wound response due, in part, to defective alveolar epithelial repair (1, 2). Various influences contribute to chronic epithelial dysfunction, including genetic factors, age, and exposure to environmental insults including cigarette smoke and viral infection (3, 4). Deficits in alveolar epithelial integrity result in compromised gas exchange, barrier dysfunction, inflammation, and excessive deposition of extracellular matrix leading to fibrosis and destruction of lung parenchyma (5, 6). The biological manifestations of disease are spatially and temporally heterogeneous, whereby normal lung tissue is adjacent to areas of fibroblast accumulation with active fibrosis or mature scar tissue with honeycomb cysts (7). Interstitial lung disease (ILD) associated with systemic sclerosis (SSc-ILD), despite arising from autoimmune vasculitis, shares many pathophysiological features in advanced stages with IPF (8). ILD, on the whole, is heterogeneous, which is reflected in the highly variable course of disease progression, and while it is well established that fibrosis in IPF and SSc-ILD is characterized by activity of myofibroblast-activating pathways such as TGF signaling, the events that lie upstream of and precipitate these pathways are not well characterized. Over the past decade, genetic studies have implicated multiple genes whose dysfunction may serve to potentiate the development of pulmonary fibrosis, which have provided insight into factors proximal to initiating/early events (9, 10).

The cellular senescence program can be induced by factors such as DNA damage, oxidative stress, mitochondrial dysfunction, endoplasmic reticulum stress, proteostasis, or dysregulated autophagy (11, 12). Senescent cells undergo cell cycle arrest and secrete proinflammatory cytokines, chemokines, proteases, and growth factors known as the senescence-associated secretory phenotype (SASP) (13, 14). SASP components recruit monocytes, macrophages, and mesenchymal cells to clear senescent cells, activate wound repair mechanisms, remodel extracellular matrix, and, under nonpathologic conditions, permit regenerative responses to restore tissue homeostasis (15, 16). When self-limited, senescence can be beneficial — e.g., during patterning morphogenesis during embryogenesis — and may serve to restrict the growth of cancerous cells (17). However, inappropriate or persistent cellular senescence can drive chronic low-grade inflammation and excessive wound repair responses, resulting in tissue fibrosis and loss of organ function (18). Increased senescent cells are observed in numerous disease states and in aging individuals (19, 20). However, precise molecular definitions and cellular origins of senescent cells in different disease states and tissue contexts are lacking.

The identification of the *MUC5B* and *SFTPC* loci as genetic risk factors implicates pulmonary epithelial dysfunction as an integral player in IPF pathogenesis (9, 21). Further studies in familial and sporadic forms of pulmonary fibrosis identified an additional group of risk alleles, including *TERT*, *TERC*, *PARN*, and *RTEL1*, all of which relate to telomere biology (22–25). Loss-of-function mutations in telomere maintenance genes implicate defective telomere maintenance in the pathogenesis of ILD, and improper maintenance or attrition of telomeres can precipitate cellular senescence. IPF patients possess significantly shorter telomeres compared with age-matched patients with other chronic diseases and exhibit increased expression of senescence markers in lung parenchyma (26–28). Taken together, these observations suggest that pulmonary epithelial cell senescence plays a role in pulmonary fibrosis pathogenesis. In preclinical mouse models, uncapping of telomeres in alveolar epithelial type II cells incurs senescence-inducing stress and drives spontaneous pulmonary fibrosis (26, 29). However, the origin and molecular phenotype of senescent cells in human ILD remain unknown. Characterization of the senescent phenotype allows for mechanistic insights into how these cells contribute to the initiation and/or progression of pulmonary fibrosis. In this study we have established in vitro models of senescent lung epithelial cells to generate a transcriptional phenotype and enable the identification of senescent alveolar epithelial cells in situ via single-cell transcriptomics from IPF and SSc-ILD lung tissue explants.

## Results

### The cellular senescence marker p16 (CDKN2A) localizes to bronchiolized epithelial structures in IPF and SSc-ILD.

**Figure 1:**
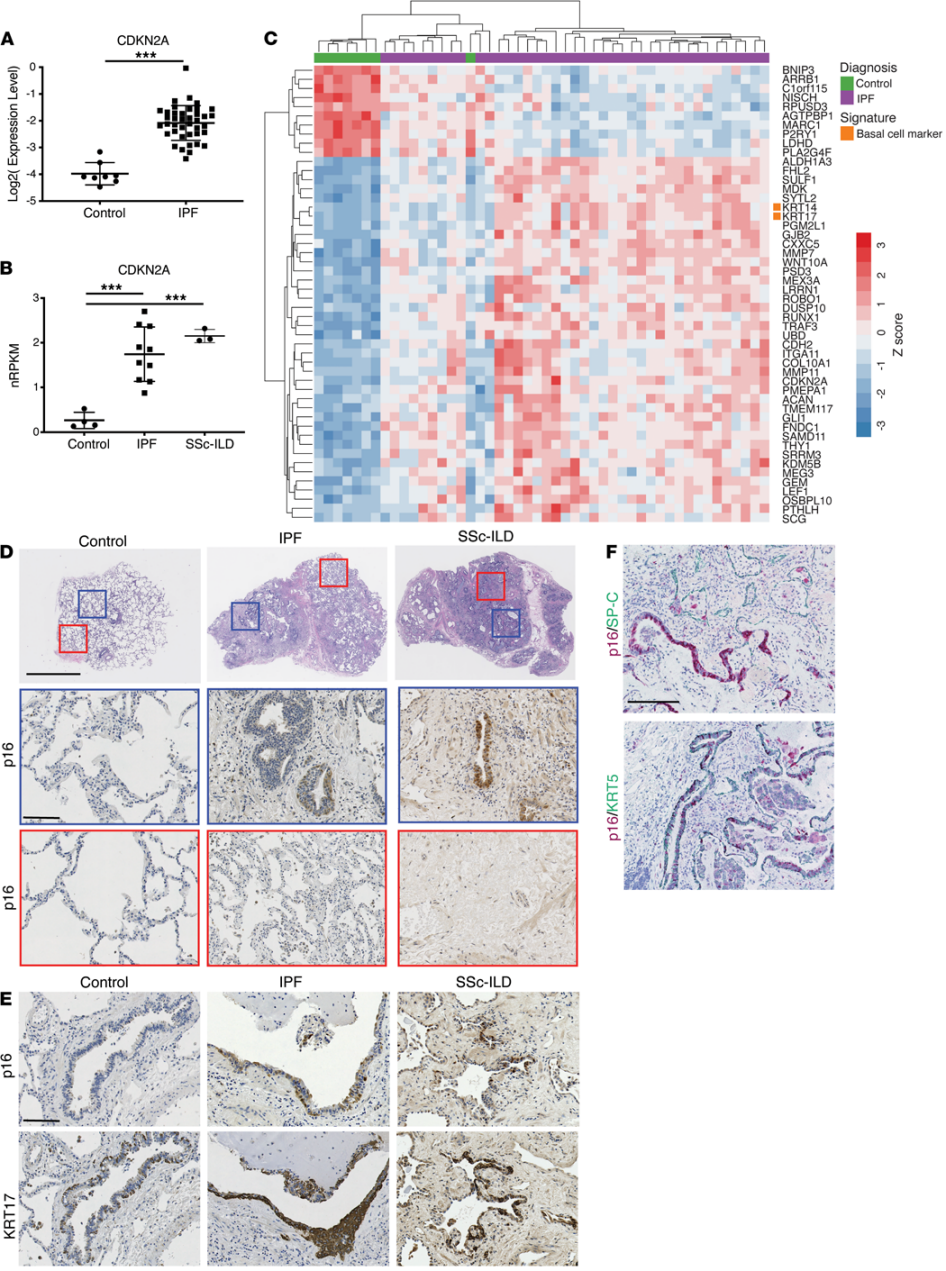
Expression of the senescent marker p16 (CDKN2A) is induced in IPF and SSc-ILD, where it localizes to basal epithelial cells in bronchiolized epithelium. (**A**) Microarray analysis of *CDKN2A* gene expression (mean ± SD, control *n* = 8, IPF *n* = 40) in control and IPF lung tissue. ***P < 0.005 (unpaired 2-tailed Student’s *t* tests). (**B**) RNA-Seq data for *CDKN2A* gene expression (mean ± SD, control *n* = 4, IPF *n* = 10, SSc-ILD *n* = 3) in control, IPF, and SSc-ILD lung tissue explants. ***P < 0.005 (Tukey’s multiple comparisons test). nRPKM, normalized reads per kilobase gene model per million total reads. (**C**) Top 50 correlates with *CDKN2A* gene expression in control and IPF lung tissue, sample set from **A**. (**D**) H&E (top row) and immunohistochemical staining of control, IPF, and SSc-ILD lung tissue sections for p16 protein. Highlighted regions from H&E shown at higher magnification from immunostainings of serial sections. (**E**) Serial sections of lung tissue stained for p16 and KRT17 proteins. (**F**) Costaining of IPF lung tissue sections for p16/SP-C and p16/KRT5 proteins. Scale bars: 200 μm (H&E), 50 μm (enlarged) (**D**); 50 μm (**E**); 100 μm (**F**).

We evaluated the cellular senescence marker p16 (*CDKN2A*), through gene expression and protein localization in the context of control, IPF, and SSc-ILD lung tissue. Consistent with previous reports (26, 27), *CDKN2A* gene expression was significantly elevated in lungs from IPF patients compared with controls (Figure 1A) from lung biopsy microarray data (Gene Expression Omnibus [GEO] GSE53845) (7). We replicated these findings in a separate cohort of patients using bulk RNA-Seq and observed a significant induction in *CDKN2A* gene expression in both IPF and SSc-ILD samples compared with controls (Figure 1B). To assess the pathobiology associated with *CDKN2A* expression, we evaluated the microarray data set for genes whose expression was highly correlated with *CDKN2A* (Figure 1C). We noted that expression of Keratin 14 (*KRT14*) and Keratin 17 (*KRT17*), specific markers for lung basal epithelial cells, were correlated with *CDKN2A*. *KRT17* also correlated with *CDKN2A* expression in the secondary set of samples (Supplemental Figure 1A; supplemental material available online with this article; https://doi.org/10.1172/jci.insight.143626DS1). Because these transcriptomic data sets were derived from bulk biopsy tissue, which we have previously demonstrated to be cytologically heterogeneous (7), we hypothesized that the increase in *CDKN2A* expression reflected an increase in senescent bronchiolar epithelial cell content in ILD samples. We next evaluated p16 protein localization by immunohistochemistry. There was substantial p16 signal in both IPF and SSc-ILD samples, specific to bronchiolized epithelial structures in scarred regions of the tissue (Figure 1D), which overlapped with the expression pattern of KRT17 in ILD. Most control samples did not register p16 signal in either alveolar or airway epithelium (Figure 1E); however, on rare occasions when p16 immunoreactivity was observed in control samples, it was in airway epithelium, not alveolar epithelium (Supplemental Figure 1B). Costaining of lung tissue sections for p16 and surfactant protein C (SP-C) or KRT5, markers of alveolar type II (ATII) or basal epithelial cells, respectively, showed that p16 expression mapped predominantly to KRT5^+^ bronchiolized epithelial structures and was not appreciably observed in SP-C^+^ ATII cells (Figure 1F). Taken together, these data suggest that basal cells within bronchiolized epithelia represent the predominant p16^+^ cell type in both IPF and SSc-ILD.

### Development of senescent lung epithelial in vitro culture models.

**Figure 2:**
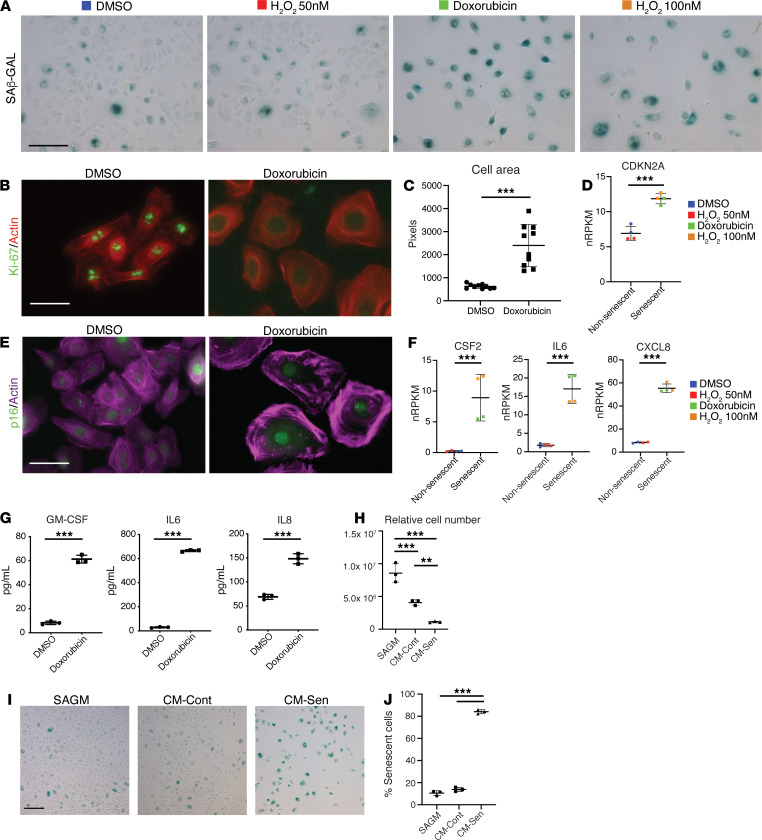
Establishing in vitro senescent lung epithelial cell models. (**A**) SAECs stained for senescence-associated β-galactosidase activity (SAβ-Gal) at 5 days after treatment (control: DMSO/H_2_O_2_ at 50 nM, senescence-inducing: doxorubicin/H_2_O_2_ at 100 nM). (**B**) Immunofluorescence (IF) staining for Ki-67 and Actin in SAECs at 5 days after treatment. (**C**) Quantification of SAEC area from representative fields (mean ± SD, *n* = 10) after induction of senescence by doxorubicin (day 5). ***P < 0.005 (unpaired 2-tailed Student’s *t* tests). (**D**) *CDKN2A* (mean ± SD, *n* = 4 biological replicates) gene expression in nonsenescent (DMSO/H_2_O_2_ 50 nM) and senescent (doxorubicin/H_2_O_2_ 100 nM) cultures at day 5. ***P < 0.005 (unpaired 2-tailed Student’s *t* tests). (**E**) IF staining for p16 and actin in SAECs at day 5 after treatment. (**F**) SASP gene expression, *CSF2*, *IL6*, and *CXCL8*, (mean ± SD, *n* = 4 biological replicates) in nonsenescent (DMSO/H_2_O_2_ 50 nM) and senescent (doxorubicin/H_2_O_2_ 100 nM) cultures at day 5. ***P < 0.005 (unpaired 2-tailed Student’s *t* tests). (**G**) Quantification of secreted SASP proteins, GM-CSF, IL-6, IL-8, (mean ± SD, *n* = 3 biological replicates) in supernatants of treated SAEC cultures over a 48-hour period (day 5 to day 7 after initiation of treatment). ***P < 0.005 (unpaired 2-tailed Student’s *t* tests). (**H**) Relative cell number in SAEC cultures at day 4 cultured in SAGM basal media, conditioned media from control cultures (CM-Cont), or conditioned media from senescent SAEC cultures (CM-Sen) (mean ± SD, *n* = 3). **P < 0.05, ***P < 0.005 (Tukey’s multiple comparisons test). (**I**) SAβ-Gal stainings of SAEC cultures at day 4. (**J**) Quantification of percentage of senescent cells in SAEC cultures treated with conditioned media at day 4 (mean ± SD, *n* = 3). ***P < 0.005 (Tukey’s multiple comparisons test). Scale bars: 100 μm (**A** and **I**), 40 μm (**B**), 40 μm (**E**).

To characterize the transcriptional phenotype of senescent lung epithelial cells in ILD, we developed in vitro models utilizing various types of primary human basal cells and methods of senescence induction. We assessed normal human bronchial epithelial cells (NHBEs) and small airway epithelial cells (SAECs) purchased commercially and freshly isolated basal epithelial cells (BECs) from control human explants. Senescence was induced via DNA damage (doxorubicin), oxidative stress (H_2_O_2_), or replicatively (serial passaging). Doxorubicin and H_2_O_2_ 100 nM–treated SAEC cultures displayed increased senescence-associated β-galactosidase activity (SAβ-Gal), with more than 90% of cells positive, compared with DMSO and H_2_O_2_ 50 nM controls, with less than 5% cells positive (Figure 2A). Senescent cells exhibited a marked reduction in protein levels of the proliferation marker Ki-67 (Figure 2B) and an enlarged morphology where cell size increased by an average of 3.85-fold (histology in Figure 2B, quantified in Figure 2C). Senescent cells induced by doxorubicin or H_2_O_2_ at 100 nM exhibited a 1.71-fold increase in *CDKN2A* (p16) gene expression compared with their control (DMSO/ H_2_O_2_ 50 nM) counterparts (Figure 2D). Senescent SAEC cultures displayed consistently stronger immunoreactivity for the p16 protein compared with DMSO controls via immunofluorescence (Figure 2E). We observed enhanced gene expression of SASP factors *CSF2*, *IL6*, and *CXCL8* in senescent SAEC cultures compared with controls (Figure 2F) (14). Coincident increases in the levels of secreted SASP proteins were observed in senescent culture supernatants (Figure 2G). Senescent cells can confer senescence on neighboring cells via SASP (30–32). To test whether senescent SAECs under the conditions tested here have that property, we cultured fresh SAECs with conditioned media from control SAEC cultures (CM-Cont) or senescent SAEC cultures (CM-Sen) and compared both of these with standard growth media (SAGM). Cell number was assessed via the CellTiter-Glo reagent measured as relative light units. At day 4, the relative cell numbers in CM-Cont media and CM-Sen media were significantly lower than in SAGM, and CM-Sen were significantly lower than CM-Cont (Figure 2H) without appreciable cell death. We assessed senescent cells via SAβ-Gal staining (Figure 2I, quantified in Figure 2J) and found that CM-Sen–treated cells were almost exclusively senescent. While there were reduced cell numbers in CM-Cont compared with SAGM, the proportions of senescent cells were comparable. These data suggest that SASP from senescent SAECs contains components that can drive bystander senescence and modulate cell fate. Having established various senescent primary human lung epithelial culture models, we performed direct comparisons to develop a consensus transcriptomic signature.

### The transcriptional phenotype of senescent cells is primarily influenced by cell type.

**Figure 3:**
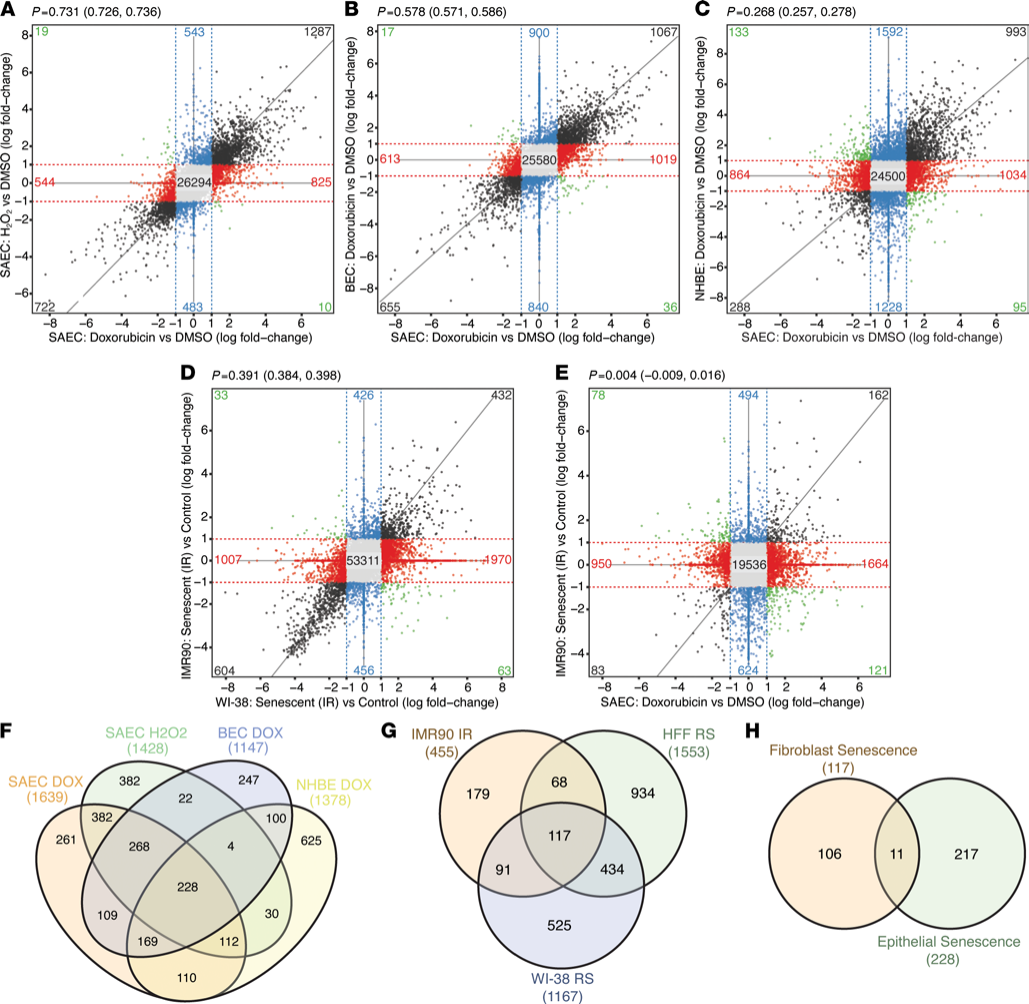
Cell type dictates senescent transcriptional phenotype. (**A**) Four-way comparison of differentially expressed genes in senescent SAEC cultures, compared with DMSO control cultures, induced by doxorubicin versus H_2_O_2_. (**B**) Four-way comparison of differentially expressed genes, compared with DMSO control cultures, in doxorubicin-treated SAEC versus doxorubicin-treated BEC cultures. (**C**) Four-way comparison of differentially expressed genes, compared with DMSO control cultures, in doxorubicin-treated SAEC versus doxorubicin-treated NHBE cultures. (**D**) Four-way comparison of differentially expressed genes, compared with control cultures, in senescent IMR90 fibroblasts induced via irradiation versus senescent WI-38 fibroblasts after replicative senescence. (**E**) Four-way comparison of differentially expressed genes, compared with control cultures, in doxorubicin-treated SAEC versus senescent IMR90 fibroblasts induced via irradiation. (**F**) Overlap in upregulated gene expression among 4 senescent lung epithelial culture models. (**G**) Overlap in upregulated gene expression among 3 senescent fibroblast models. (**H**) Overlap between the consensus senescent lung epithelial signature (**F**) and consensus senescent fibroblast epithelial signature (**G**). IR, irradiation-induced senescence; RS, replicative senescence. (**A–E**) *P* = Spearman’s correlation, 95% confidence intervals in parentheses.

The transcriptional phenotypes of senescent cells were highly intercorrelated (Spearman’s rho *P* = 0.268–0.731) across all epithelial culture models regardless of induction method. Senescence induction in SAEC cultures, whether by doxorubicin or H_2_O_2_, produced a consistent phenotype where differentially expressed genes were highly intercorrelated (rho = 0.731) (Figure 3A). Comparison of differential gene expression between senescent cultures of SAECs and BEC/NHBE, induced via doxorubicin, showed correlations of 0.578 and 0.268, respectively (Figure 3, B and C). The senescent phenotype proved stable over time or when cultured on different substrates (Supplemental Figure 2, A and B). The phenotypes of senescent fibroblast cultures induced via irradiation (IMR90) or serial passaging (WI-38), derived from public data sets GSE94395 (33) and GSE63577 (34), showed a similar intercorrelation with each other (rho = 0.391) as we observed within senescent lung epithelial cultures (Figure 3D). However, the transcriptional changes in senescent SAECs and senescent IMR90 lung fibroblasts (GSE94935) were not correlated (rho = 0.004, Figure 3E), demonstrating that fibroblasts and epithelial cells exhibit distinct transcriptional changes upon senescence induction.

We derived a consensus epithelial senescence gene signature by identifying the overlap between 4 senescent lung epithelial data sets and selecting genes within each data set based on the following criteria: a fold change in expression ≥ 2 between treatment and control, a Bonferroni-corrected *P* ≤ 0.05, and an average log_2_ nRPKM ≥ 1.0 (overlap between conditions shown in Figure 3F). This yielded a total of 228 coregulated senescence-associated genes shared among the different epithelial cell types (Supplemental Table 1). Of note, *CDKN2A* did not appear in this signature as induction was not consistent across models (data not shown), but p16 protein levels and activity can be further regulated posttranscriptionally (35, 36). We employed a similar approach to generate a consensus signature for senescent fibroblasts, serving as a comparator to epithelial senescence (Figure 3G and Supplemental Table 2). Overlap between the epithelial and fibroblast consensus signatures was limited to 11 genes (Figure 3H, Supplemental Figure 2C, and Supplemental Table 3), none of which has been previously described as a senescence marker. These findings suggest that cell type of origin strongly influences the transcriptional phenotype of a senescent cell. For that reason, we developed a senescent signature from BECs because they exhibited features of senescence in IPF and SSc-ILD (Figure 1), hypothesizing that it would have greater potential to identify and characterize senescent cells in fibrotic lung tissue in situ.

### The in vitro–derived senescent lung epithelial signature overlaps with a replicative senescence signature in a single-cell RNA-Seq data set.

**Figure 4:**
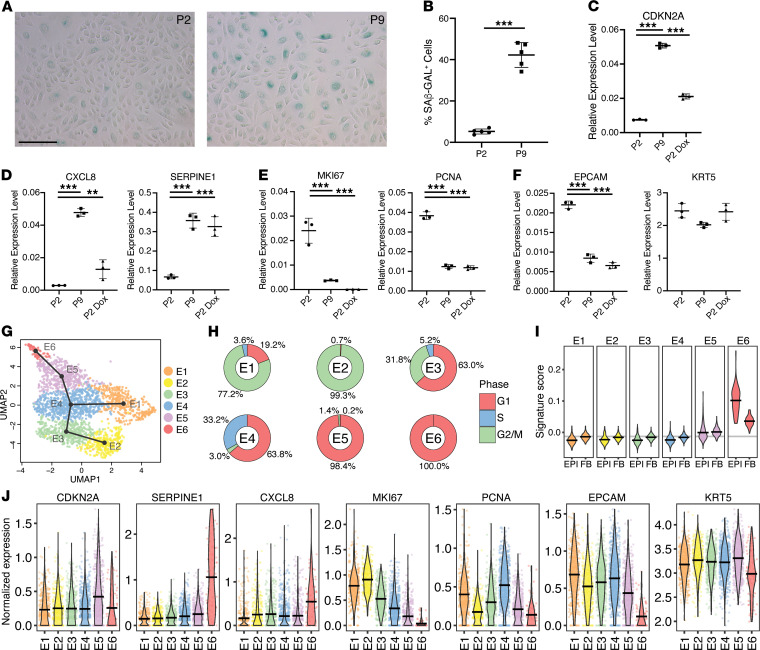
Consensus senescence signature identifies replicative senescence in primary human lung epithelial cell cultures. (**A**) SAβ-Gal staining of BEC cultures at passages 2 and 9. (**B**) Percentage of SAβ-Gal^+^ cells (mean ± SD, *n* = 5 biological replicates) in BEC cultures at passages 2 and 9. ***P < 0.05 (unpaired 2-tailed Student’s *t* tests). (**C**) *CDKN2A* gene expression (mean ± SD, *n* = 3 biological replicates) in BEC cultures at P2 and P9 and P2 doxorubicin-treated cultures. ***P < 0.05 (Tukey’s multiple comparisons test). (**D**–**F**) Gene expression (mean ± SD, *n* = 3 biological replicates) of SASP, proliferation markers, and basal cell markers in BEC cultures at P2 and P9 and P2 doxorubicin-treated cultures. **P < 0.05, ***P < 0.005 (Tukey’s multiple comparisons test). (**G**) Uniform manifold approximation and projection (UMAP) plot showing scRNA-Seq cluster assignments in BEC culture at P4, with the overlaid black lines showing inferred trajectory between clusters as a minimum spanning tree with cluster E2 as the starting point. (**H**) Distribution of cells in each BEC culture cluster that were annotated as being in the G1, S, or G2/M phase of the cell cycle. (**I**) Violin plots showing signature scores of BEC culture clusters for both the epithelial-derived (EPI) and fibroblast-derived (FB) senescence-associated gene sets; the black horizontal bar denotes the mean expression value within the given cluster. (**J**) Violin plots showing the normalized expression of select senescence- and proliferation-associated genes in each BEC culture cluster; each point represents an individual cell, and the black horizontal bar denotes the mean expression value within the given cluster. Scale bars: 100 μm (**A**).

Serial passaging of primary human lung basal cells results in increased numbers of cells undergoing replicative senescence. The percentage of SAβ-Gal^+^ cells increased from 5.25% at passage 2 to 42.3% at passage 9 (Figure 4, A and B), coincident with increased expression of *CDKN2A* and the SASP factors *SERPINE1* and *CXCL8* (IL-8) (Figure 4, C and D) and reduced proliferation markers *PCNA* and *MKI67* (Figure 4E). Expression of the basal cell marker *KRT5* was not significantly altered by serial passaging, while expression of the epithelial marker *EPCAM* was significantly reduced in later passages (Figure 4F). Transcriptional effects of serial passaging were directionally consistent with changes observed in doxorubicin-treated cultures (Figure 4, C–F). We sought to identify a senescent subset of cells within an intermediate passage (P4) by single-cell RNA-Seq (scRNA-Seq), in which 8.82% of cells were SAβ-Gal^+^. Figure 4G displays a UMAP plot from the P4 primary lung BEC culture scRNA-Seq data, illustrating 6 epithelial populations after clustering was performed. Inferred trajectory between clusters as a minimum spanning tree is overlaid onto the clustering in the UMAP plot. This shows that cluster E4 is a branching point from which trajectories diverge. Cells were annotated as being in G1, S, or G2/M phases of the cell cycle based on expression of established markers (Figure 4H). Clusters E2, E5, and E6 almost exclusively comprised cells in a single-cell cycle stage, with clusters E5 and E6 annotated as G1 and E2 annotated as G2/M. Senescent cells having undergone cell cycle arrest remain in G1; hence we hypothesized that either cluster E5 or E6 might represent senescent cells.

We next evaluated the expression of epithelial cell–derived and fibroblast-derived senescence gene signatures described in Figure 3. Senescence signature scores were calculated for each cell by subtracting the mean normalized expression of 100 random control genes from the mean normalized expression of the signature gene sets (37). Therefore, a higher signature score, above 0, translates to an enrichment in induced gene expression of members of this gene set built on senescence-specific induced gene expression. The epithelial signature score identified cluster E6 as the sole population displaying a significant enrichment of a senescent transcriptional phenotype (Figure 4I), which displayed an association, albeit weaker, with the fibroblast-derived senescent signature. While cluster E6 does not exhibit enhanced *CDKN2A* mRNA expression, there is a distinct upregulation of senescence-associated genes *CXCL8* and *SERPINE1*, accompanied by downregulation of proliferation-related genes *PCNA* and *MKI67* (Figure 4J). When examining prototypical basal cell markers, the expression of *KRT5* was consistent across clusters, while *EPCAM* expression was significantly lower in cluster E6 (Figure 4J), as observed in later passages and in doxorubicin-treated cultures. The cells comprising cluster E6 were almost exclusively annotated as being in G1. Interestingly the cluster E5 population largely lay in G1, exhibited an induction of *CDKN2A* gene expression and lowered *MKI67* and *PCNA* gene expression, and appeared as a transitional population in terms of trajectory from cluster E5 to cluster E6. This cluster showed a slight elevation of the senescence signature score. This population could potentially capture a “presenescent” or quiescent population. Therefore, the consensus epithelial senescence signature derived in Figures 2 and 3 demonstrates the capacity to identify a discrete senescent subpopulation in a heterogeneous mixture of epithelial cells, which was enhanced compared with the fibroblast-derived senescence signature.

### IPF and SSc-ILD are enriched for a senescent BEC population.

**Figure 5:**
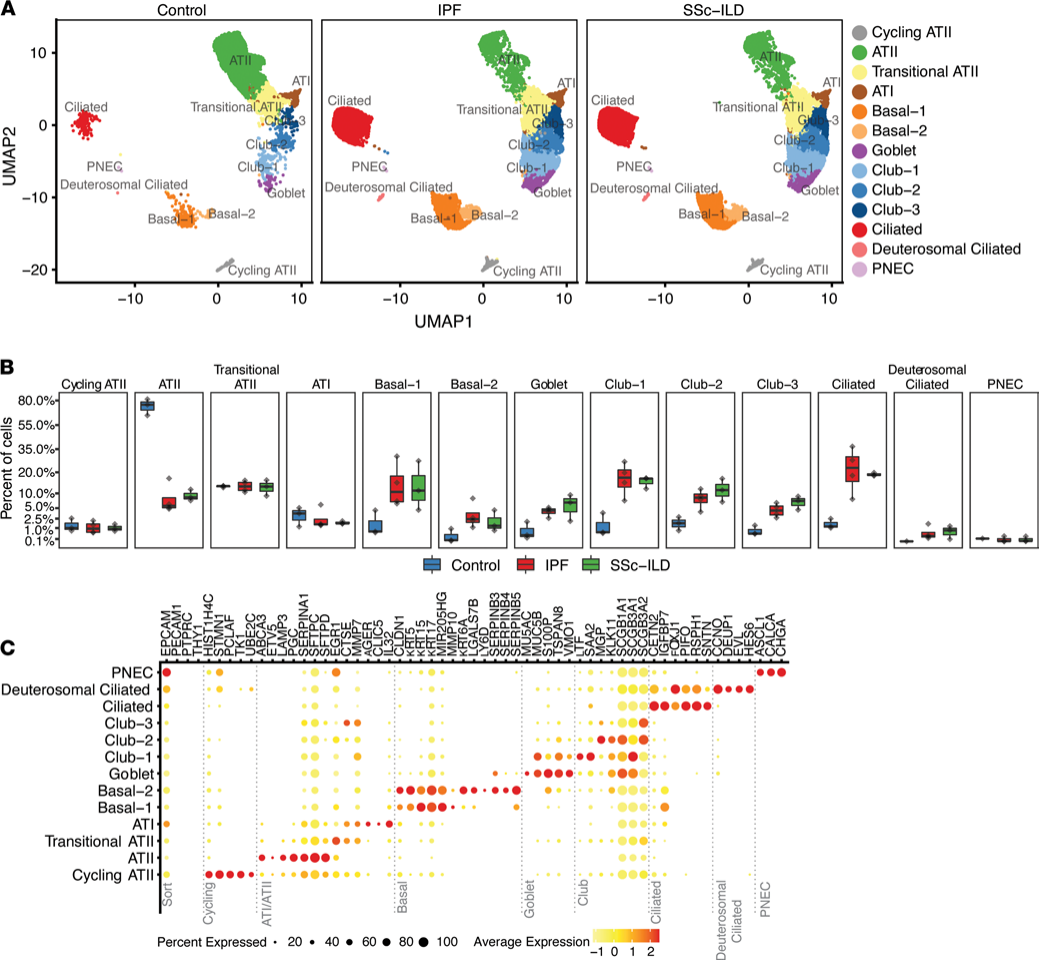
Characterization of the epithelial populations in IPF and SSc-ILD. (**A**) UMAP plot of epithelial cell populations from human lung explants after clustering. PNEC, pulmonary neuroendocrine cells. (**B**) Percentage of cells in each cluster shown as individual boxplots for the control, IPF, and SSc-ILD samples; *y* axis is on a square root scale. The box plots depict the minimum and maximum values (whiskers), the upper and lower quartiles, and the median. The length of the box represents the interquartile range. (**C**) Dot plot of selected canonical cell type markers and marker genes identified by across-cluster differential expression; color (yellow to red) denotes mean normalized expression for the cluster; dot size denotes the percentage of cells within the cluster for which any expression was detected, with no dot shown for percentage detected values under 15%.

We sought to determine the relationship between the in vitro senescence signatures and cell populations within primary human ILD tissue. We dissociated explanted lung tissue from control, IPF, and SSc-ILD patients to create a single-cell suspension from which live cells were sorted for scRNA-Seq. Patient demographic information is listed in Supplemental Table 4. Epithelial cells were captured as the CD45^–^CD31^–^EpCAM^+^ population from single-cell suspension and prepared for sequencing using the 10x Genomics Chromium platform. Figure 5A displays a UMAP plot of 13 epithelial cell clusters identified by scRNA-Seq after removal of nonepithelial cell clusters. There was a notable shift in the proportions of epithelial cell types between control and fibrotic samples (IPF, SSc-ILD), in which ATI/II populations were reduced in pulmonary fibrosis. Conversely, airway epithelial cells, including all basal, ciliated, and secretory populations, were markedly enriched in fibrotic samples (Figure 5B). Cell type annotations were assigned using the expression of a combination of established marker genes and between-cluster differential expression (Figure 5C).

**Figure 6:**
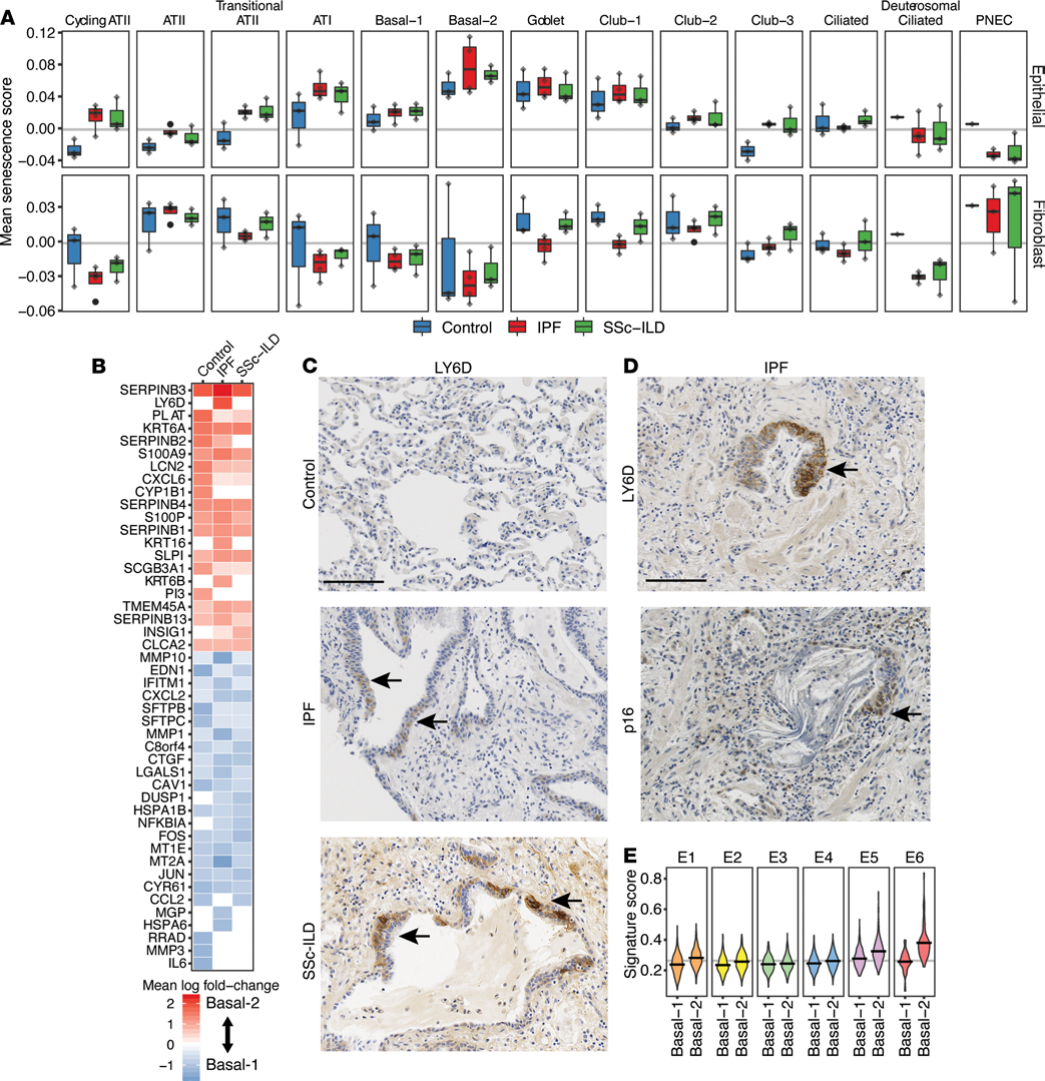
Identification of a senescent BEC population enriched in the fibrotic lung. (**A**) Boxplots showing the distribution and mean signature scores for both epithelial cell–derived and fibroblast-derived consensus senescence gene sets; each point reflects the mean signature score for an individual subject within the given cluster. The box plots depict the minimum and maximum values (whiskers), the upper and lower quartiles, and the median. The length of the box represents the interquartile range. (**B**) Heatmap showing the log fold change in normalized expression between the Basal-1 and Basal-2 populations for genes in the union set of control, IPF, and SSc-ILD differentially expressed genes across these 2 clusters; genes upregulated in Basal-2 are shown in red, and genes upregulated in Basal-1 are shown in blue. (**C**) IHC staining of control, IPF, and SSc-ILD lung tissue sections for LY6D. (**D**) IHC staining of IPF lung tissue serial sections for LY6D and p16. (**E**) Scoring of clusters E1–6, from Figure 4, against Basal1/2 gene expression signatures. Scale bars: 100 μm (**C**), 40 μm (**D**).

We looked for senescent epithelial populations in the same manner as the scRNA-Seq data from primary lung epithelial cell cultures described in Figure 4. We observed the most robust enrichment of the epithelial cell–derived senescence signature in the Basal-2 population, followed by the Goblet and Club-1 populations (Figure 6A), which were all more abundant in, but not unique to, IPF and SSc-ILD samples (Figure 6B). Meanwhile, no epithelial population showed a significant enrichment for the fibroblast senescence signature. The Basal-2 cluster was of particular interest as the expression pattern of the senescence marker p16 was shown to predominantly localize to KRT17^+^ BECs in bronchiolized structures in fibrotic lung, but scarcely in airway BECs in control samples (Figure 1, D–F).

In comparison with all epithelial clusters, the Basal-2 population is distinguished by elevated expression of *LY6D*, *KRT6A*, *SERPINB3/4/5*, and *LGALS7B* (Supplemental Table 5). Of these, *LY6D*, *KRT6A*, and *SERPINB3* are part of the consensus epithelial senescence signature. The top 20 most differentially regulated genes in the control, IPF, and SSc-ILD samples between the Basal-1 and Basal-2 clusters are shown in Figure 6B as a heatmap of the mean log fold change of normalized expression values. Many markers that distinguish the Basal-2 cluster from the epithelial populations as a whole continued to distinguish this population when compared directly with another basal cell population, Basal-1. Induction of *CDKN2A* gene expression was not observed in this population, which is aligned with the lack of clear and consistent transcriptional induction previously described in in vitro senescence models and which may reflect a difference between transcript and protein levels (35, 36), considering the immunoreactivity for p16 protein observed in Figure 1. Notably, there was lower expression of genes associated with regulation of both apoptosis and senescence (*LGALS1*, *CAV1*, *FOS*, *JUN*, *CYR61*) in the Basal-2 cluster (Figure 6B). In cells encountering stress, such as DNA damage, the balance between senescent and apoptotic signaling often decides cell fate (38, 39), and there appears to be complex contextual interplay between these programs that requires further investigation. A *SERPINB3*^+^*LY6D*^+^ basal epithelial population, equivalent to the Basal-2 cluster, can be observed within 3 previously published independent scRNA-Seq data sets (Supplemental Figure 3) (40–42). The Basal-2 population is clearly distinct from the ILD-associated epithelial populations described by Adams et al. and Habermann et al. as aberrant basaloid or *KRT5*^–^*KRT17*^+^, which reside within the ATI cluster of our data set. Markers for these populations are specifically induced in a proportion of ATI cells from IPF and SSc-ILD but not control samples (Supplemental Figure 4). This discrepancy in clustering of populations that are transcriptionally similar to ATI cells, but express a set of basal cell markers, may arise due to the different computational batch correction and sample integration methods employed.

We evaluated the localization of LY6D, a defining Basal-2 cluster marker, by immunohistochemistry in control, IPF, and SSc-ILD lung samples (Figure 6, C and D). No signal for LY6D protein was detected in control alveolar or airway tissue. Immunoreactivity was specific to bronchiolized structures in IPF and SSc-ILD tissue in a pattern similar to that of p16, displaying overlap in serial sections. These data provide evidence for a senescent basal cell population that to our knowledge has not been previously described, is significantly expanded in fibrotic lung tissue, and can be identified by surface LY6D expression. To compare the features of this cluster with our in vitro senescence model, we used the differential gene expression defining the Basal-1 and Basal-2 clusters as signatures to score enrichment within clusters E1–E6 from the BEC cultures in Figure 4 (Figure 6E). The Basal-2 signature was clearly enriched in cluster E6, which we defined as senescent via orthogonal analyses. In addition, cluster E2, which we had proposed as a potential presenescent population, showed a slight enrichment for the Basal-2 signature. Therefore, there is consistency between senescent phenotypes in BECs in multiple in vitro culture models and senescence in vivo.

### Senescent BECs in ILD enact a p53-dependent squamous differentiation gene expression program.

**Figure 7:**
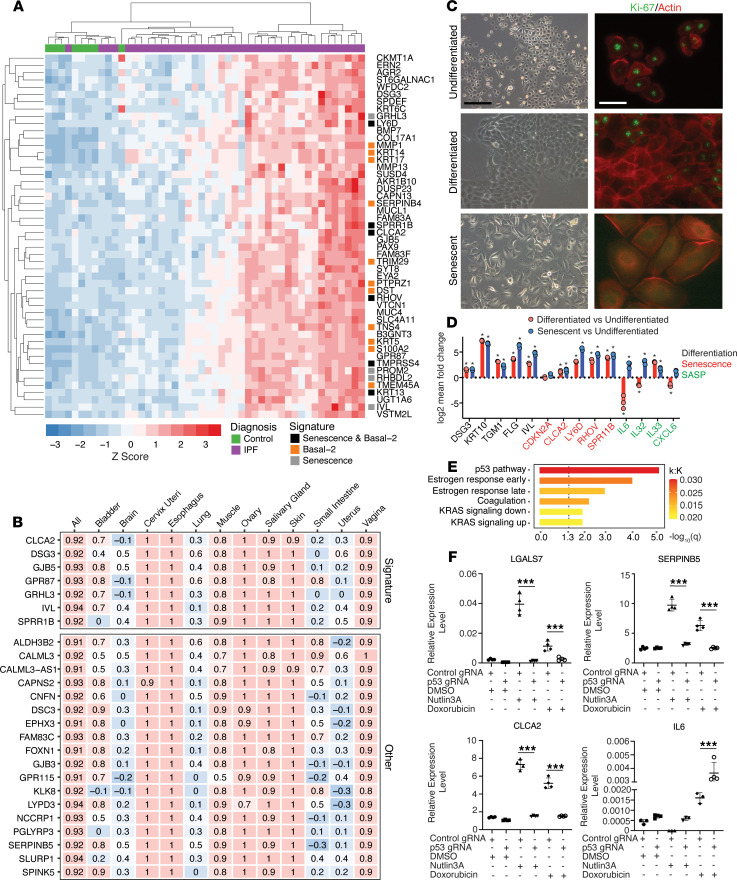
Basal cell senescence is transcriptionally related to squamous terminal differentiation. (**A**) Top correlates with *LY6D* gene expression in lung tissue from control and IPF patient explants. (**B**) Top correlates with *LY6D* across various tissues. (**C**) Phase images of normal human epidermal keratinocyte (NHEK) cultures under basal, differentiation-inducing, and senescence-inducing culture conditions (on left) and IF staining for Ki-67 in NHEK cultures in proliferating, differentiated, and senescent cultures (on right). (**D**) Comparison of differential gene expression between terminally differentiated keratinocytes and senescent keratinocytes compared with undifferentiated cultures (mean ± SD, *n* = 3). *P < 0.05 (ratio of means *t* test). (**E**) GSEA Hallmark pathway enrichment in the Basal-2 population based on differential gene expression with associated FDR *q* values and overlap enrichment k:K (# of overlapping genes: # of genes in pathway signature). (**F**) Gene expression of squamous and SASP markers in gene-edited NHBE cultures at 24 hours (mean ± SD, *n* = 3). ***P < 0.005 (unpaired 2-tailed Student’s *t* test). Scale bars: 100 μm (phase in **C**), 40 μm (IF in **C**).

To extend the characterization of the senescent basal epithelial population in ILD lung tissue, we identified transcripts highly correlated with *LY6D* in control and IPF lung tissue (GSE53845) (Figure 7A). As expected, these included BEC markers *KRT5*, *KRT14*, and *KRT17*. In addition, we observed substantial overlap with the consensus in vitro–derived senescent epithelial signature and the differentially expressed genes specifically enriched in the Basal-2 population from scRNA-Seq profiling (Figure 7A). To identify potential biological correlates with the ILD senescent basal cell signature and infer function, we evaluated whether this signature was conserved in other tissue types utilizing the expression data available on the GTEx portal (https://gtexportal.org/home/). The genes in the *LY6D*-related signature were highly correlated in a subset of tissues, including cervix/uteri, esophagus, ovary, skin, and vagina (Figure 7B). Intriguingly, many of these tissues are characterized by stratified squamous epithelia in which a mitotically active basal epithelial layer is overlaid with epithelial cells undergoing terminal differentiation. Coincidentally, *LY6D* expression in skin is restricted to terminally differentiating suprabasal cells (43). To further probe the transcriptional relationship between basal cell senescence and terminal differentiation, we examined cultured primary human epidermal keratinocytes.

Culturing in low-calcium media (0.03 mM) maintains keratinocytes in an undifferentiated state. Elevated levels of calcium (2.8 mM) in culture media induce terminal differentiation (44). We compared differentially expressed genes between low and high calcium (terminal differentiation) or doxorubicin treatment in low calcium (senescence). Each culture condition resulted in distinct keratinocyte morphology (Figure 7C). Undifferentiated cells appeared rounded, without extensive contacts to neighboring cells. Terminally differentiated cells exhibited extensive contact with one another and displayed a typical cobblestone appearance. Senescent keratinocytes treated with doxorubicin were enlarged and flattened, similar to in vitro senescent lung BECs (Figure 2, B and E). Ki-67 immunofluorescence exhibited reduced signal in both the differentiated and senescent cultures, consistent with exit from the cell cycle (Figure 7C).

Transcriptomic analysis displayed an extensive overlap between differential gene expression in senescent and terminally differentiated cells (Supplemental Figure 5). In both cases, compared with undifferentiated cells, senescent and terminally differentiated cells exhibited downregulation of genes associated with proliferation (Supplemental Figure 5) and upregulation of genes associated with differentiation, as well as upregulation of genes associated with basal epithelial senescence (Figure 7D). Serial passaging of primary keratinocytes leads to increased replicative senescence and a pattern of gene expression similar to that observed via doxorubicin-induced senescence (data not shown). A key transcriptional difference between senescent and terminally differentiated keratinocytes was the expression of SASP factors, which are induced in senescent cultures but generally reduced or unchanged upon terminal differentiation (Figure 7D). These data suggest that withdrawal from the cell cycle due to terminal differentiation or senescence exhibits overlapping transcriptional phenotypes. This may account for the increased senescent signature scores observed in the Goblet and Club-1 clusters in lung (Figure 6A), as these are terminally differentiated cells in which particular aspects may overlap with a senescent program, yet the squamous differentiation-related gene expression aspect of the senescent phenotype appears to be a distinguishing feature (Supplemental Figure 6).

We performed pathway analysis using gene set enrichment analysis (GSEA) on the top differentially expressed genes (fold change > 1.5) from the Basal-2 cluster (Supplemental Table 5). Pathways showing enrichment within this set of 43 genes are listed in Figure 7E. The p53 pathway, which displayed the most significant enrichment, has been shown to play an active role in both differentiation and senescence (45–49). A similar analysis with a gene set from the replicatively senescent cluster E6 population from Figure 4 also demonstrated an enrichment in the p53 signaling pathway (data not shown). We investigated the role of p53 in senescence of human bronchiolar BECs via pharmacological manipulation and gene editing. Chronic p53 activation, via treatment with Nutlin-3a, was sufficient to induce target gene expression and cell cycle arrest leading to senescence in NHBE cultures (Supplemental Figure 7, A–C). The absence of p53, established by way of CRISPR gene targeting, in the context of senescence-inducing genotoxic stress via doxorubicin treatment, resulted in apoptosis rather than senescence (Supplemental Figure 7, D–G). Additionally, the induction of squamous differentiation-related genes that define senescent lung BECs, demonstrated by the exemplars *LGALS7*, *SERPINB5*, and *CLCA2*, was driven by p53 (Figure 7F). In contrast, the SASP marker *IL6* was not induced by Nutlin-3a but was induced by doxorubicin treatment in control-targeted cells. The induction of *IL6* observed by doxorubicin treatment was accentuated in p53-targeted cultures. These results demonstrate that squamous differentiation-related gene expression is directly modulated by p53, whereas SASP components may be induced via a p53-independent mechanism or blunted by p53 activation, consistent with published studies (50).

## Discussion

Cellular senescence can be a beneficial adaptation in various contexts, including development, tissue repair/regeneration, and tumor suppression (18). In nonpathogenic contexts, senescent cells persist for a limited time, eventually cleared by immune cells recruited via cytokines and chemokines secreted by the senescent cells. However, many studies have shown that senescent cells accumulate with age where their prolonged presence is negatively associated with health and life span (51); timing and context ultimately dictate whether effects are beneficial or detrimental. An illustrative example of this dual nature of cellular senescence is wound repair. In the liver, stellate cell senescence promotes tissue healing and regeneration while limiting fibrotic responses; however, specific telomere shortening and senescence in hepatocytes limits regenerative capacity and correlates with fibrotic progression (52, 53). Induced senescence of cardiac fibroblasts and ATII cells promotes heart and lung fibrosis, respectively (29, 54). In human pulmonary fibrosis, there are still many questions that remain unanswered: what constitutes the senescent cell population; what effect(s) do they have on surrounding cells; how do they exert these effects; can senescent cells be selectively eliminated, and if so, what are the consequences of eliminating senescent cells? The advent of single-cell transcriptomics has been instrumental in mapping discrete cell populations in the lung and can enable identification and characterization of subpopulations of cells, such as senescent cells, and provide insights into how these cells contribute to disease pathogenesis.

In the present study, we showed that in IPF and SSc-ILD lung tissue the putative senescent marker p16 is predominantly localized to bronchiolized epithelium lining honeycomb cysts, specifically to KRT17^+^ basal cells. We established in vitro models of senescent lung BECs and generated a consensus transcriptional signature to identify senescent cell populations in situ. It has become apparent that, much as senescence can be beneficial or maladaptive depending on context, senescent phenotypes are variable and contextual across tissues and cell types (55). For both fibroblasts and BECs, the transcriptomes of senescent cells were well correlated within a given cell type, independent of induction method. In contrast, the transcriptional phenotype of senescent BECs and senescent fibroblasts showed little overlap. The 11 induced genes that overlapped between the consensus epithelial and fibroblast signatures (Supplemental Table 3) contained genes such as *CCND2*, a cell cycle regulator; *WDR63* and *HIST1H1C*, both known to interact/influence p53 signaling; *C1QTNF1*, identified as an mTOR regulator; and the long noncoding RNA *FER1L4*, which has been shown to regulate phosphatase and tensin homolog. Therefore, several markers in the 11-gene overlap relate to cell cycle or proliferation-related signaling, which is consistent with cells withdrawing from the cell cycle. However, the vast majority of induced genes in senescent epithelial cells and fibroblasts were divergent; thus, the senescent phenotype is cell type specific, highlights the need to model senescence in a relevant system because markers may not translate across settings, and highlights the lack of an established universal marker(s) of senescence and a need to develop complementary and more expansive tools to explore this phenomenon. This is especially true in complex cellular contexts relevant to disease pathogenesis, such as profiling cells from primary tissue in scRNA-Seq experiments.

We used the consensus epithelial senescence signature to identify a population undergoing replicative senescence in an unbiased clustering of scRNA-Seq data from primary human lung basal epithelial cultures. This signature differentiated a single cluster enriched for gene expression induced in senescent culture models. In contrast, the fibroblast senescence signature did not convincingly identify a potentially senescent population, underscoring the value of a targeted senescent model system. We then used the consensus epithelial senescence signature to probe an scRNA-Seq data set generated from control, IPF, and SSc-ILD lungs harvested at explant, specifically focused on the epithelial compartment.

The epithelial senescence signature distinguished several epithelial clusters as enriched for a senescent transcriptional phenotype. These included the Basal-2 cluster, a population of BECs highly enriched in, but not exclusive to, IPF and SSc-ILD. These findings align with the pattern of p16 expression, which colocalizes to KRT17^+^ BECs in bronchiolized epithelia. p16^+^ cells in airway epithelium of control samples are likely due to replicative senescence observed in aged individuals. Cluster markers defining this senescent basal cell population correlate with one another in transcriptional analysis of IPF lung tissue via bulk RNA-Seq, providing evidence of a coherent signal preserved at the tissue level. Our findings identify a senescent basal cell population that is not defined by canonical senescence markers, but rather show specific enrichment for previously undocumented senescent lung epithelial cell–related gene expression patterns. Importantly, the Basal-2 population, in part defined by *SERPINB3*^+^ and *LY6D*^+^ markers, is detectable in several recently published scRNA-Seq studies (40–42).

To develop hypotheses about the cellular processes that may be contributing to the unique phenotype of senescent BECs in ILD, we looked for the unique signatures of these cells in primary lung tissue and evaluated whether they overlap with signals in other tissues. Interestingly, markers associated with senescent lung BECs aligned with a conserved transcriptional module present in multiple stratified squamous epithelial tissues. *IVL*, *LY6D*, and *CLCA2* are all expressed in a differentiation-specific manner in the epidermis and other squamous epithelia (43, 56). CLCA2 has also been implicated in promoting senescence downstream of p53 (57). LY6D and CLCA2 are cell surface markers that can be used to distinguish and isolate senescent lung BECs or potentially provide value as noninvasive biomarkers in peripheral blood, as each can be cleaved from the cell surface, capturing the overall burden of lung epithelial senescence (58, 59). Further studies should examine whether these proteins are detectable as systemic biomarkers associated with disease progression in patients with ILD, as has been shown for another marker of this senescent population, SERPINB3 (60). Other senescence-related biomarkers, such as telomere length and GDF15, along with MMP3, a marker specific to bronchiolized epithelium where senescent cells reside, have been shown to be associated with more advanced disease and/or rapid disease progression in patients with fibrotic disorders (7, 25, 61).

Our observations suggest that there is a convergence in the transcriptional phenotype of epithelial cells that have exited the cell cycle via either senescence or terminal differentiation. This is also reflected in the enrichment of the senescence signature in terminally differentiated Goblet and Club-1 clusters. Scoring of clusters from our scRNA-Seq data set for a signature composed of squamous terminal differentiation-related genes showed a more specific enrichment within the Basal-2 cluster (Supplemental Figure 6). This implicates the squamous differentiation aspect of the Basal-2 population as a distinguishing feature of the senescent phenotype in lung epithelium.

GSEA revealed evidence of p53 pathway activation in the Basal-2 population. We also noted p53-inducible transcripts in in vitro–derived epithelial (*TP53I3*) and fibroblast (*TP53INP1*) consensus signatures (Supplemental Tables 1 and 2). The p53 pathway can play an active role in the execution of senescent, apoptotic, and differentiation programs. Activation of the p53 pathway, alone or in the context of cellular/genotoxic stress, in lung BECs, is necessary and sufficient to promote senescence, while coincidentally inducing squamous differentiation-related gene expression. Several of the squamous markers observed in senescent cells — *CLCA2*, *SERPINB5*, and *LGALS7* — possess p53 response elements in their proximal promoters (62–64). *GRHL3*, another member of the squamous module present in the consensus signature that is induced by p53 activation (data not shown), has been shown to play an active role in keratinocyte differentiation (65). While these data suggest a potential relationship between the p53 pathway and terminal differentiation in squamous epithelia, p53-deficient mice do not exhibit overt defects in squamous differentiation; loss, rather than activation, of p53 is linked to squamous differentiation (66–68).

A distinguishing feature of senescence is the induction of SASP. We show that the SASP from senescent SAEC cultures can directly drive bystander senescence in proliferating SAEC cultures; however, the individual SASP component(s) responsible for this effect remain to be determined. Several immune modulators observed in SASP of senescent lung BEC cultures, including IL-6, IL-8, and GM-CSF, are known to act on monocytes/macrophages. Consistent with this notion, monocyte-derived macrophages are recruited into tissues with chronic pathological fibrosis, where they play an active role in disease pathogenesis (69). We further observed that p53 induces terminal differentiation markers but is not required for *IL6* induction (Figure 7), consistent with prior reports (50). Our context-specific in vitro senescence models provide an avenue to characterize SASP, gain insight into how this population of cells acts to modulate disease pathogenesis, and identify potential context-specific targets for therapeutic intervention.

Recent studies have touted the use of senolytic agents targeting the elimination of senescent cells for therapeutic treatment of IPF (70). While the elimination of senescent cells via senolytics would remove potential detrimental effects of SASP, whose net effects remain unknown at this point, it may come with negative effects. In a background where there is ample regenerative capacity, elimination of senescent cells may not be problematic, but this is not the case in the fibrotic lung. In this instance, the alveolar epithelial stem cell compartment, ATII cells, is unable to regenerate and reestablish integrity because of senescence. Removal of epithelial cells may have a feed forward effect whereby the epithelium is further compromised, while advancing additional cells into a senescent state that could potentially accelerate fibrosis. A more nuanced approach to targeting senescent cells or their biological effects may be warranted.

## Methods

### Cell culture.

NHBEs and SAECs were purchased from Lonza and cultured in PneumaCult-EX Plus media (STEMCELL Technologies, 05040) and SAGM (Lonza, CC-3118), respectively. For IMR90 and WI-38 fibroblasts, data were obtained from public data sets GEO GSE94395 and GSE63577, respectively, and source information can be found associated with these data sets and/or corresponding manuscripts. BECs were isolated directly from human lung explants — see below for sample preparation — and the single-cell suspension was plated and cultured in SAGM to propagate expansion of basal epithelial cultures, followed by enrichment/purification with CD326 (EpCAM) microbeads (Miltenyi Biotec, 130-061-101).

Epithelial cell cultures were treated with doxorubicin, at 500 nM, for 5 days, whereupon cells were then harvested for RNA isolation, fixed for immunofluorescence staining, or stained for SAβ-Gal expression. Supernatants from senescent cultures were collected at day 7, 48 hours after media exchange on day 5, and examined for the secretion of immunomodulatory molecules. Control cultures treated with DMSO were harvested along a similar timeline. Cultures were treated with 50 or 100 nM of H_2_O_2_ for 1 hour, then washed 3 times with PBS, followed by subsequent culture for downstream RNA isolation, collection of culture supernatant, immunofluorescence staining, and SAβ-Gal staining.

RNA isolation and SAβ-Gal staining were performed on serially passaged BEC cultures to investigate replicative senescence in primary lung epithelial cells. BEC cultures at passage 4 were utilized in an scRNA-Seq experiment.

### Conditioned media experiments.

Conditioned media from control, DMSO, and doxorubicin-treated cultures were collected after cells had been subjected to treatment for 5 days. After a media exchange, the cultures were incubated for 48 hours, and cell culture supernatant was collected and cleared by centrifugation at 300*g* for 5 minutes, at room temperature. Conditioned media used to treat SAEC cultures was formulated by combining SAGM and conditioned media in a 1:1 ratio. SAEC cultures were treated for 2 days, followed by an exchange to refresh the conditioned media, and evaluations were performed at day 4 after treatment.

### CRISPR gene targeting.

CRISPR knockout mutations were generated as described (71) with slight modifications. Specifically, individual single guide RNAs (sgRNAs) and Cas9 Electroporation Enhancer (IDT, 1075916) were each resuspended at 100 μM in nuclease-free duplex buffer (IDT, 11-01-03-01). Individual sgRNAs were pooled at equimolar ratio at 100 μM final total concentration. A ribonucleoprotein (RNP) complex was prepared by mixing pooled sgRNAs, HiFi Cas9 Nuclease (IDT, 1081061), and Cas9 Electroporation Enhancer at final concentration of 77 μM, 7.7 μM, and 7.7 μM, respectively. Nuclease-free duplex buffer was added to achieve total RNP volume 14.4 μL. The RNP complex was gently pipetted 5 times and left at room temperature for 30 minutes. Freshly expanded UNCN1T cells (72) (Kerafast, ENC011) were harvested using Tryp-LE Express (Gibco, Thermo Fisher Scientific, 12604013) and resuspended in P3 buffer from P3 Primary Cell 4D-Nucleofector X Kit (Lonza, V4XP-3032) at 2.22 × 10^7^ cells/mL. Then, 10.8 μL of cell suspension was mixed with 14.4 μL of RNP solution and pipetted gently twice, and 21 μL was transferred into a well of Nucleocuvette strip. Electroporation was performed using CM-113 setting on 4D-Nucleofector (Lonza, AAF-1002B) with X Unit (Lonza, AAF-1002X). Electroporated cells were transferred into 2 T25 flasks, each containing 5 mL of PneumaCult-Ex Plus Medium (STEMCELL Technologies, 05040) and 10 μM Y-27632 (AdipoGen, AGCR13564M025).

### Immunohistochemistry.

Formalin-fixed, paraffin-embedded lung tissue sections were cleared and rehydrated in Target Retrieval Solution, Citrate pH 6.1 (Dako) heated to 95°C and cooled for 1.5 hours. Then, sections were subjected to a 5-minute PBS wash and a 10-minute incubation in peroxidase/alkaline phosphatase blocking solution (BLOXALL, Vector Laboratories). Samples were washed for 5 minutes in PBS and blocked for 20 minutes in Animal-Free Blocker (Vector Laboratories). Tissue sections were incubated in primary antibodies diluted in 1× Animal-Free Blocker at 4°C overnight. This was followed by three 5-minute PBS washes and a 30-minute incubation in secondary antibody, Powervision Poly-HRP anti-rabbit or anti-mouse (PV6119, PV6114, Leica Biosystems). After three 5-minute PBS washes, samples were stained by DAB reagent ImmPACT DAB EqV (Vector Laboratories). Samples were washed in tap water, counterstained with hematoxylin, dehydrated, and mounted. For IHC costaining, immunohistochemistry on formalin-fixed, paraffin-embedded tissue was performed on the Ventana immunostaining platform. Images were taken on a Zeiss Axio Imager M2 microscope.

### Immunofluorescence.

Cells were fixed with 4% formaldehyde in PBS for 15 minutes, washed 3 times for 5 minutes in PBS, then blocked and permeabilized with a 1× PBS/5% normal goat serum/0.3% Triton X-100 solution for 1 hour at room temperature. Primary antibodies were diluted in a 1× PBS/1% BSA/0.3% Triton X-100 solution and incubated overnight at 4°C. Slides were washed 3 times with PBS and incubated in secondary antibody (A10520, A10521, Thermo Fisher Scientific) for 1 hour at room temperature. Samples were stained with DyLight 488 phalloidin (12935S, Cell Signaling Technology), washed 2 times for 5 minutes with PBS, and mounted with DAPI-containing ProLong Gold Antifade Mountant (Thermo Fisher Scientific). Images were taken on a Zeiss Axio Imager M2 microscope.

### Antibodies.

We used KRT5 (PRB-160P, Covance Biologicals), KRT17 (AB183330, Abcam), Pro-SPC (AB3796, Chemicon), p16 (E6H4, Roche Tissue Diagnostics, 705-4793), Ki67 (D3B5, Cell Signaling Technology), and LY6D (PA5-64167, Thermo Fisher Scientific).

### Bulk RNA-Seq library preparation and sequencing.

Total RNA was isolated using the RNeasy kit (Qiagen) per manufacturer’s suggestions. Quality control of total RNA was done to determine sample quantity and quality. The concentration of RNA samples was determined using NanoDrop 8000 (Thermo Fisher Scientific), and the integrity of RNA was determined by Fragment Analyzer (Advanced Analytical Technologies). A total of 0.1 μg of RNA was used as input material for library preparation using TruSeq Stranded Total RNA Library Prep Kit (Illumina). Size of the libraries was confirmed using 4200 TapeStation and High Sensitivity D1K screen tape (Agilent Technologies), and concentration was determined by quantitative PCR–based method using library quantification kit (KAPA). Libraries were multiplexed and sequenced on Illumina HiSeq4000 (Illumina) to generate 30 million single-end 50–base pair reads.

### Bulk RNA-Seq analysis library preparation and sequencing.

Sequencing reads were filtered and aligned using HTSeqGenie v4.2.2 (73). GSNAP v2013-11-01 was used for alignment, through the HTSeqGenie wrapper, against the GENCODE Basic gene model on the human genome assembly GRCh38. Only reads with unique genomic alignments were analyzed. nRPKM values were used as a normalized measure of gene expression, calculated as previously defined in Srinivasan et al. 2016 (74). Log_2_ nRPKM transformations were calculated on nRPKM+1^–4^, and the *z*-scored log_2_ nRPKM ranges displayed in the heatmaps were restricted to ±3 standard deviations of the log_2_ nRPKM values for visualization purposes; heatmap clustering was performing using Euclidean distance and complete linkage. Differential gene expression was calculated using voom+limma (75) with multiple-hypothesis correction of *P* values performed using the Benjamini-Hochberg method. RNA-Seq data were deposited in the National Center for Biotechnology Information’s (NCBI) GEO database (accession GSE166059).

### Preparation of human lung tissue.

After bronchoalveolar lavage, fresh lung explant tissue was stored in complete media on wet ice overnight. The tissue was washed in HBSS and thoroughly minced in digestion buffer (HBSS, 2.5 mg/mL collagenase D, 100 μg/mL DNAse). Minced tissue was rocked 45 minutes at 37°C. Residual tissue material was transferred into fresh digestion buffer and rocked another 45 minutes at 37°C. Single cells from both rounds of digest were combined and utilized for downstream analyses. Single-cell preparations were labeled with a cocktail of fluorescently labeled antibodies, including CD45 (BD Biosciences, 563879/clone HI30), EpCAM (BD Biosciences, 347198/clone EBA-1), CD31 (BD Biosciences, 563651/clone WM59), CD90 (BD Biosciences, 559869/clone SE10), and Live/Dead (eBioscience, Thermo Fisher Scientific 65-0865-14), then subjected to fluorescence-activated cell sorting (FACS) to isolate specific populations.

### Single-cell RNA-Seq library preparation and sequencing.

Single-cell RNA-Seq was performed on the 10x Genomics platform using Chromium Single Cell 3′ library and gel bead kit v2 following manufacturer’s user guide (10x Genomics). In brief, the cell density and viability of single-cell suspension were determined by Vi-CELL XR cell counter (Beckman Coulter). All samples had high percentage of viable cells. The cell density was used to impute the volume of single-cell suspension needed in the reverse transcription master mix, aiming to achieve approximately 6000 cells per sample. cDNAs and libraries were prepared following manufacturer’s user guide (10x Genomics). Libraries were profiled by Bioanalyzer High Sensitivity DNA kit (Agilent Technologies) and quantified using Kapa Library Quantification Kit (Kapa Biosystems). Each library was sequenced in 1 lane of HiSeq4000 (Illumina) following manufacturer’s sequencing specification (10x Genomics). RNA-Seq data were deposited in the NCBI’s GEO database (accession GSE159354).

### Single-cell RNA-Seq analysis.

Sequencing reads were assembled and aligned against the GRCh38 human reference using Cell Ranger v3.1.0 (10x Genomics). Expression count matrices were analyzed using the Seurat v3.1 (37) R package (76). Only cells with at least 500 features and no more than 5% total mitochondrial feature counts were retained for analysis. Normalization was performed using the log normalization method. Approximately 4000 highly variable features were selected using the mean/variance regression method for sample integration and clustering after removal of immunoglobulin and T cell receptor variable domain features from the highly variable feature set. Sample integration and batch correction were performed using the anchor-based sample integration workflow for the control, IPF, and SSc-ILD lung tissue samples (37); this step was not performed for the primary cell culture experiment. Clustering was performed using 20 principal components analysis components on a k = 20 shared nearest neighbor (SNN) graph using the Louvain algorithm. UMAP dimensional reductions were performed using umap-learn v0.3.10 (77) on the same SNN graph used for clustering. Marker selection was performed using Wilcoxon’s rank-sum test on the integrated sample data for each cluster against all clusters. Cell type annotations were performed manually based on differentially expressed markers meeting the criteria of having an average log fold change compared with all other clusters of at least 0.8; being either detected (detection defined as having at least 1 unique molecular identifier for the feature) in at least 50% of the cells in a given cluster or detected in no more than 10% of all other clusters; and showing no clear evidence of ambient RNA contamination, which necessarily excluded some classical markers, such as *SFTPC*, *SCGB1A1*, *SCGB3A1*, and *SCGB3A2*, among others. Cells were then pared down to only epithelial cells for further analysis. Trajectory inference for the cell culture scRNA-Seq data was performed using slingshot v1.4.0 (78) with the UMAP coordinates as input and cluster E2 designated as the starting point.

### Luminex.

Cytokine levels were assayed by Luminex technology using MILLIPLEX MAP kits from MilliporeSigma according to manufacturer’s protocol. Fluorescence intensities (FIs) from the labeled beads were read using a FlexMaps instrument from Luminex Corp. FIs from diluted standards were used to construct standard curves using Bio-Plex Manager software from Bio-Rad Laboratories using either 4-pl or 5-pl regression type. Data are presented as average of 3 samples run in duplicate measurements.

### SAβ-Gal staining.

Staining was performed with the Senescence Detection Kit (Abcam) per manufacturer’s protocol. Cells were fixed for 15 minutes at room temperature with 1× fixation solution, washed with PBS, and then stained overnight at 37°C in 1× staining solution. Bright-field images were captured on the Zeiss Axio Imager M2 microscope.

### Quantitative PCR.

A total of 200 ng of RNA input was utilized in the reverse transcription reaction (High-Capacity cDNA Reverse Transcription Kit, Thermo Fisher Scientific). cDNA was utilized in standard quantitative PCR reaction utilizing TaqMan assays (Thermo Fisher Scientific, probes listed in Supplemental Table 6) for specific gene targets and TaqMan Universal PCR Master Mix. Amplification was run on the QuantStudio 6 Real-Time PCR System (Applied Biosystems, Thermo Fisher Scientific). Data were normalized to the mean of housekeeping genes *HPRT1* and *TFRC*.

### Statistics.

Data are expressed as the mean ± SD in the main text and figures. All experiments were repeated 2 or more times. Statistical analyses were done with Prism 8 (GraphPad Software). Variable differences between experimental groups were assessed using the 2-tailed Student’s *t* test and Tukey’s multiple comparisons test. A *P* value less than 0.05 was considered significant. Confidence intervals for Spearman’s correlation statistics were calculated by *z* transformation. *P* values for the ratio of means were calculated using Student’s *t* test assuming mutually independent Gaussian errors (79). Biological replicates were conducted using different lots of cultured cells.

### Study approval.

Explanted lung tissues were obtained from patients with a pathologic diagnosis of usual interstitial pneumonia and a consensus clinical diagnosis of IPF assigned by multidisciplinary discussion and review of clinical materials or from patients with SSc-ILD who met American College of Rheumatology criteria for scleroderma (80). Written informed consent was obtained from all subjects, and the study was approved by the UCSF institutional review board. Human lungs not used by Donor Network West were used as controls; studies indicate that these lungs are physiologically and pathologically normal (81).

## Author contributions

DJD designed, performed, and analyzed experiments and drafted the manuscript. JAVH analyzed experiments and assisted in drafting the manuscript. KBM, KS, and ZM assisted in the scRNA-Seq studies. GT performed NHBE culture experiments. PJW provided human lung tissue and scientific insight. JRA supervised the study and reviewed and edited the manuscript.

## Supplementary Material

Supplemental data

Supplemental Table 5

## Version 1 03/11/2021

In-Press Preview

## Version 2 04/22/2021

Electronic publication
