# Supplementary material for: Molecular mapping of interstitial lung disease reveals a phenotypically distinct senescent basal epithelial cell population
Source: JCI Insight. 2021 Apr 22;6(8):e143626. doi: 10.1172/jci.insight.143626 (PMC8119199; doi:10.1172/jci.insight.143626)
Supplement: Supplemental data [file jciinsight-6-143626-s026.pdf]

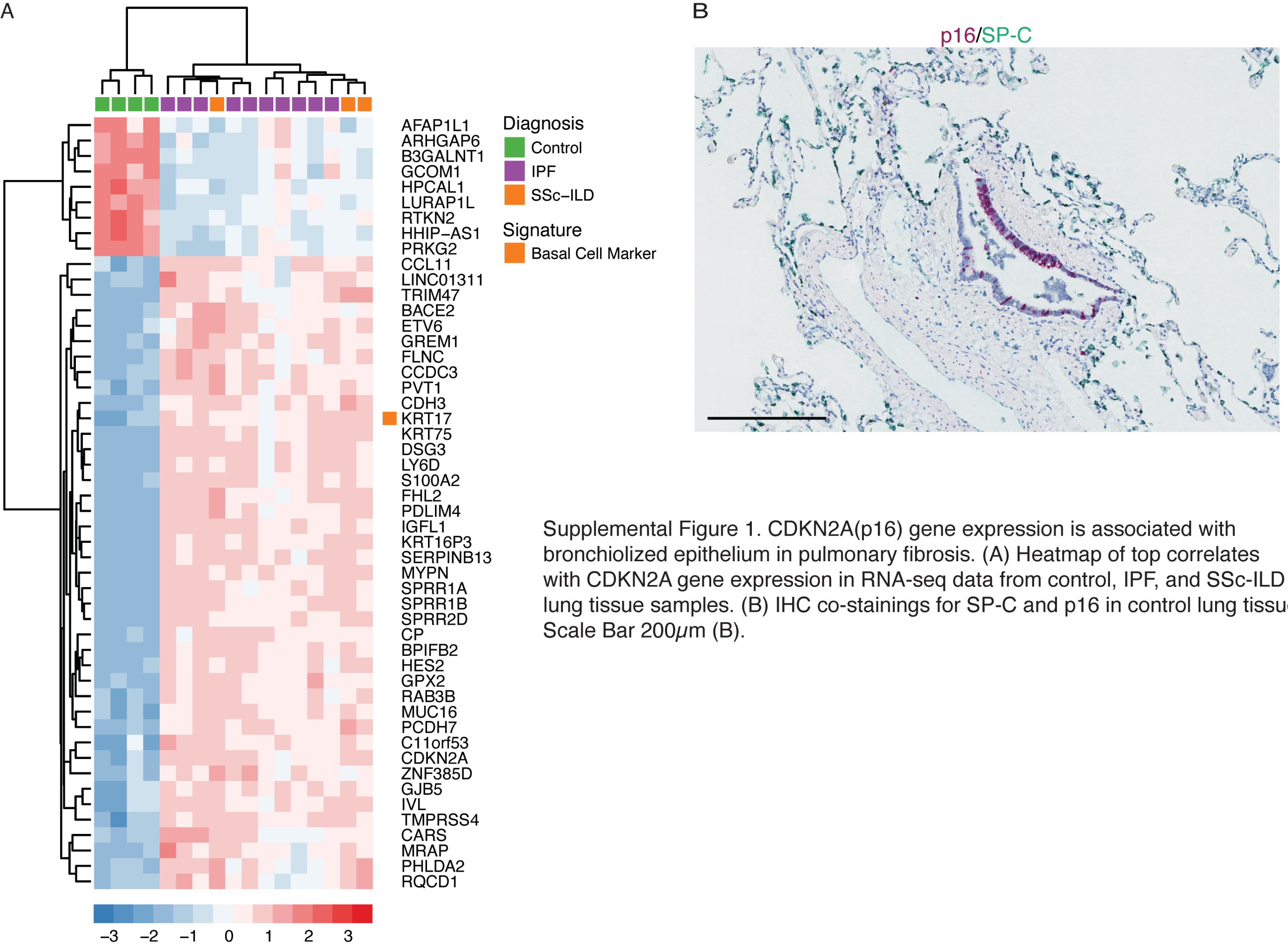

Supplemental Figure 1. CDKN2A(p16) gene expression is associated with bronchiolized epithelium in pulmonary fibrosis. (A) Heatmap of top correlates with CDKN2A gene expression in RNA-seq data from control, IPF, and SSc-ILD lung tissue samples. (B) IHC co-stainings for SP-C and p16 in control lung tissue. Scale Bar 200μm (B).

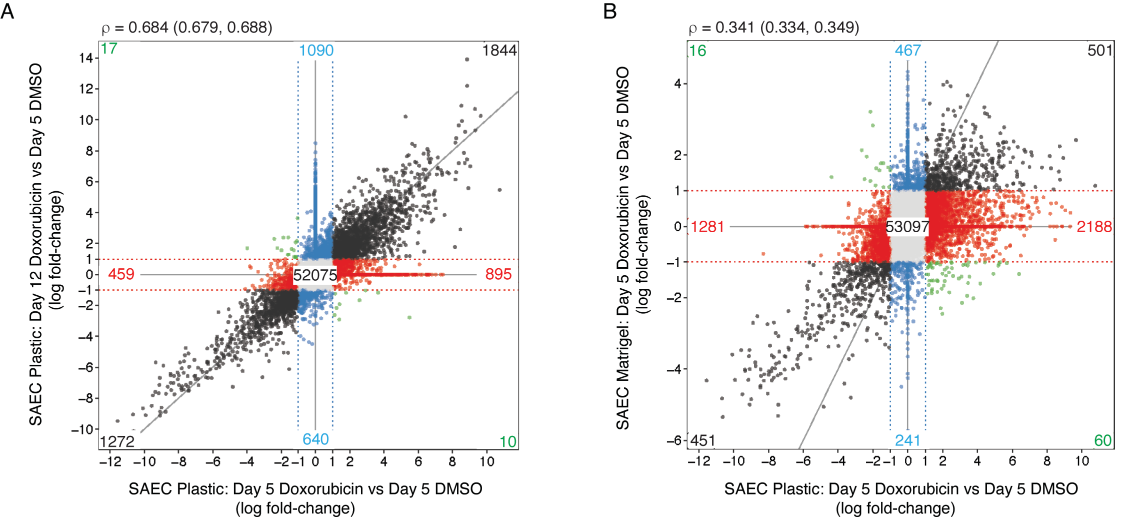

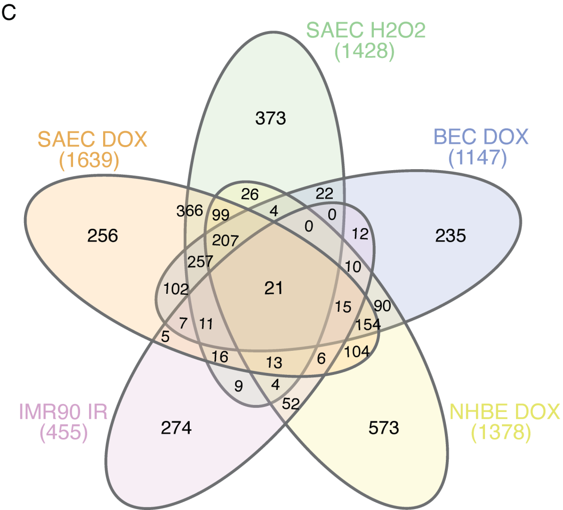

Supplemental Figure 2. Senescent phenotype is stable and is cell type dependent. (A) 4-way comparison of differential gene expression in senescent SAEC cultures at day 5 versus day 12 after treatment. (B) 4-way comparison of differential gene expression in senescent SAEC cultures on plastic versus matrigel at day 5. (C) Overlap between transcriptional phenotype of senescent epithelial culture models and senescent fibroblast model (IMR90).

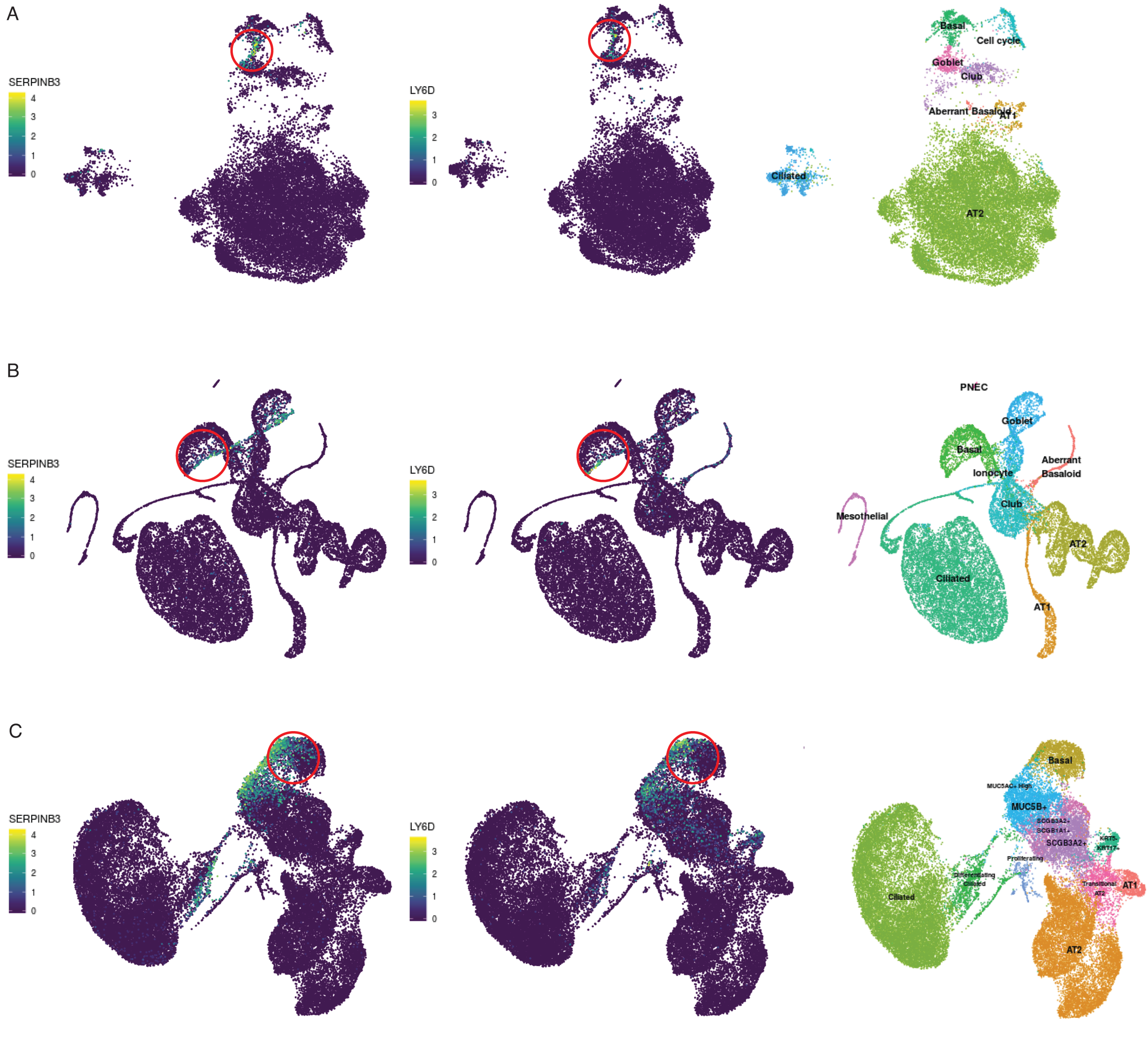

Supplemental Figure 3. Basal-2 population present in independent scRNA-seq data sets. (A) UMAP plot demonstrating *SERPINB3* and *LY6D* expression co-localized to designated basal cells in Reyfman et al data set (33) generated on the IPF Cell Atlas website. (B) UMAP plot demonstrating SERPINB3 and LY6D expression co-localized to designated basal cells in the Adams et al., (35) data set generated on the IPF Cell Atlas website. (C) UMAP plot demonstrating SERPINB3 and LY6D expression co-localized to designated basal cells in the Habermann et al., (34) data set generated on the IPF Cell Atlas website.

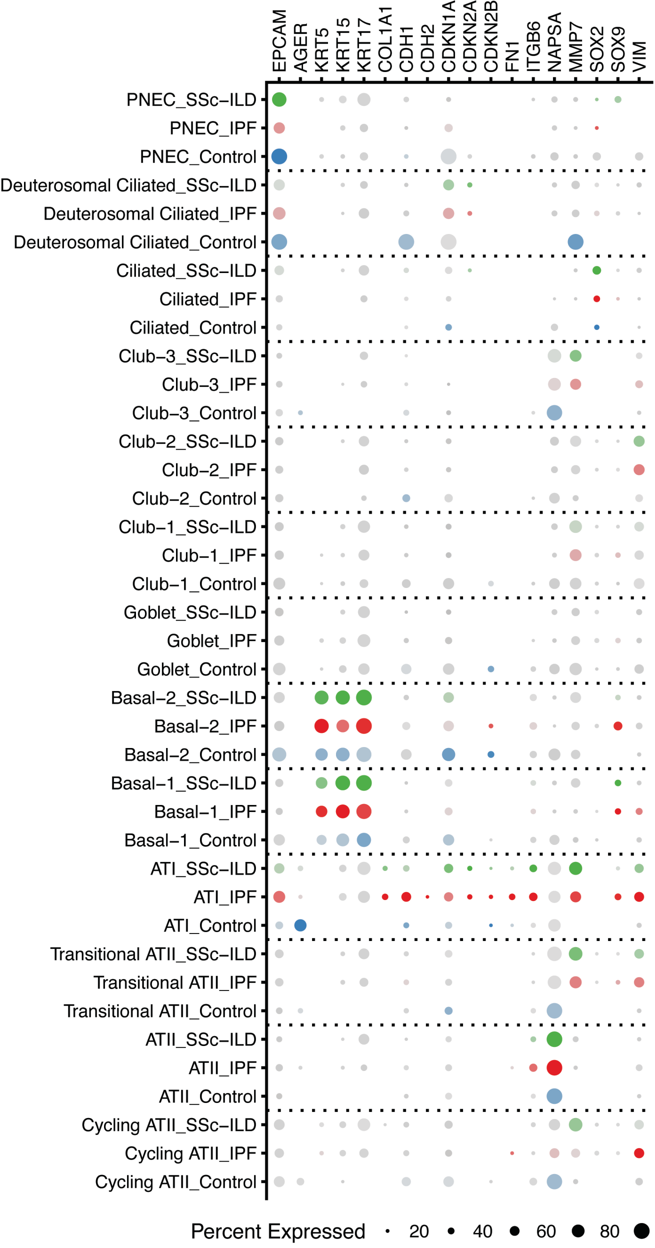

Supplemental Figure 4. Expression of Aberrant Basaloid and KRT5-/KRT17+ markers across epithelial clusters. Dotplot showing the average expression and percent detected of select markers defining the aberrant basaloid and KRT5-/KRT17+ epithelial cell populations described by Adams et al and Habermann et al across cell population and diagnosis.

A

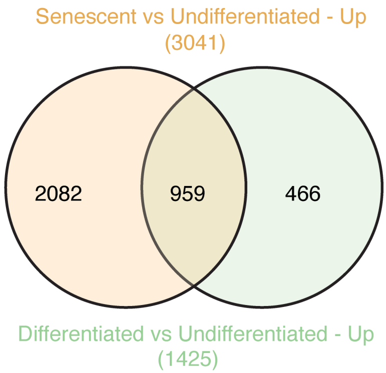

B GO Biological Process (FDR)

<u>Senescent only</u>

Membrane depolarization (3.62E-02)
Regulation of regulated secretory pathway (4.88E-03)
Regulation of exocytosis (2.42E-03)
Extracelular matrix organization (2.91E-02)
Localization within membrane (4.22E-02)

<u>Both Senescent and DIfferentiation</u>

Establishment of skin barrier (4.24E-06)
Cornification (7.81E-31)
Keratinization (1.14E-21)
Multicellular organismal water homeostasis (4.29E-06)
Keratinocyte differentiation (4.01E-24)

<u>Differentiation only</u>

Fatty acid elongation, unsaturated fatty acid (2.85E-02)
Fatty acid elongation, saturated fatty acid (2.78E-02)
Connective tissue development (4.59E-02)
Epithelial cell migration (1.18E-02)
Fatty-acyl-CoA biosynthetic process (3.38E-02)

C

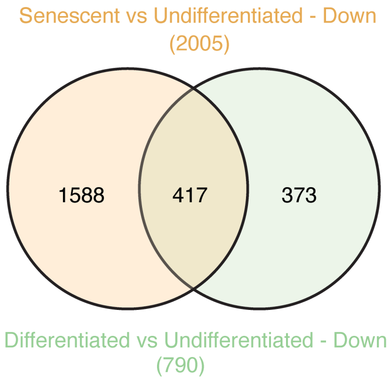

D GO Biological Process (FDR)

<u>Senescent only</u>

microtubule nucleation by microtubule organizing center (8.27E-03)
DNA ligation involved in DNA repair (2.50E-02)
purine nucleobase biosynthetic process (2.49E-02)
nucleobase biosynthetic process (2.48E-02)
DNA strand elongation involved in DNA replication (3.27E-02)

<u>Both Senescent and DIfferentiation</u>

Strand invasion(1.47E-02)
Double-strand break repiar via break-induced replication(6.17E-06)
Regulation of mitochondrial ATP synthesis coupled electron transport(3.09E-02)
Positive regulation of exit from mitosis(3.08E-02)
Pre-replicative complex assembly involved in cell cycle DNA replication(4.64E-03)
Mitotic spindle midzone assembly(4.59E-03)

<u>Differentiation only</u>

positive regulation of cell adhesion (8.06E-03)
regulation of cell adhesion (2.40E-03)
cytokine-mediated signaling pathway (4.70E-02)
response to cytokine (1.29E-02)
cellular response to cytokine stimulus (2.19E-02)

Supplemental Figure 5. Comparison of senescence and differentiation transcriptional profiles. (A) Overlap of upregulated differential gene expression, FC > 2, $p < 0.05$, between senescent and differentiated keratinocytes. (B) List of top 5 enriched GO Biological processes, with false discovery rate (FDR), forgene sets upregulated in senescent cells only, shared between senescent and differentiated cells, and differentiated cells only. (C) Overlap of down-regulated differential gene expression, FC > 2, $p < 0.05$, between senescent and differentiated keratinocytes. (D) List of top 5 enriched GO Biological processes, with false discovery rate (FDR), for gene sets down-regulated in senescent cells only, shared between senescent and differentiated cells, and differentiated cells only.

A

Squamous terminal differentiation signature genes

LY6D	IVL	SPRR1B
GRHL3	CALML3	NCCRP1
GPR87	CALML3-AS1	RHOV
DSC3	CLCA2	
DSG3	LYPD3	

B

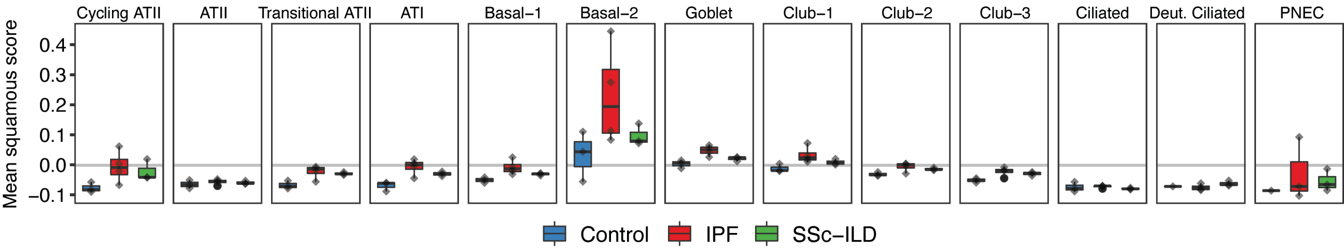

Supplemental Figure 6. Squamous differentiation gene expression associated with basal cell senescence is a distinct feature that distinguishes the Basal-2 population. (A) List of genes comprising squamous terminal differentiation signature. (B) Scoring of individual clusters to gauge enrichment of squamous terminal differentiation signature.

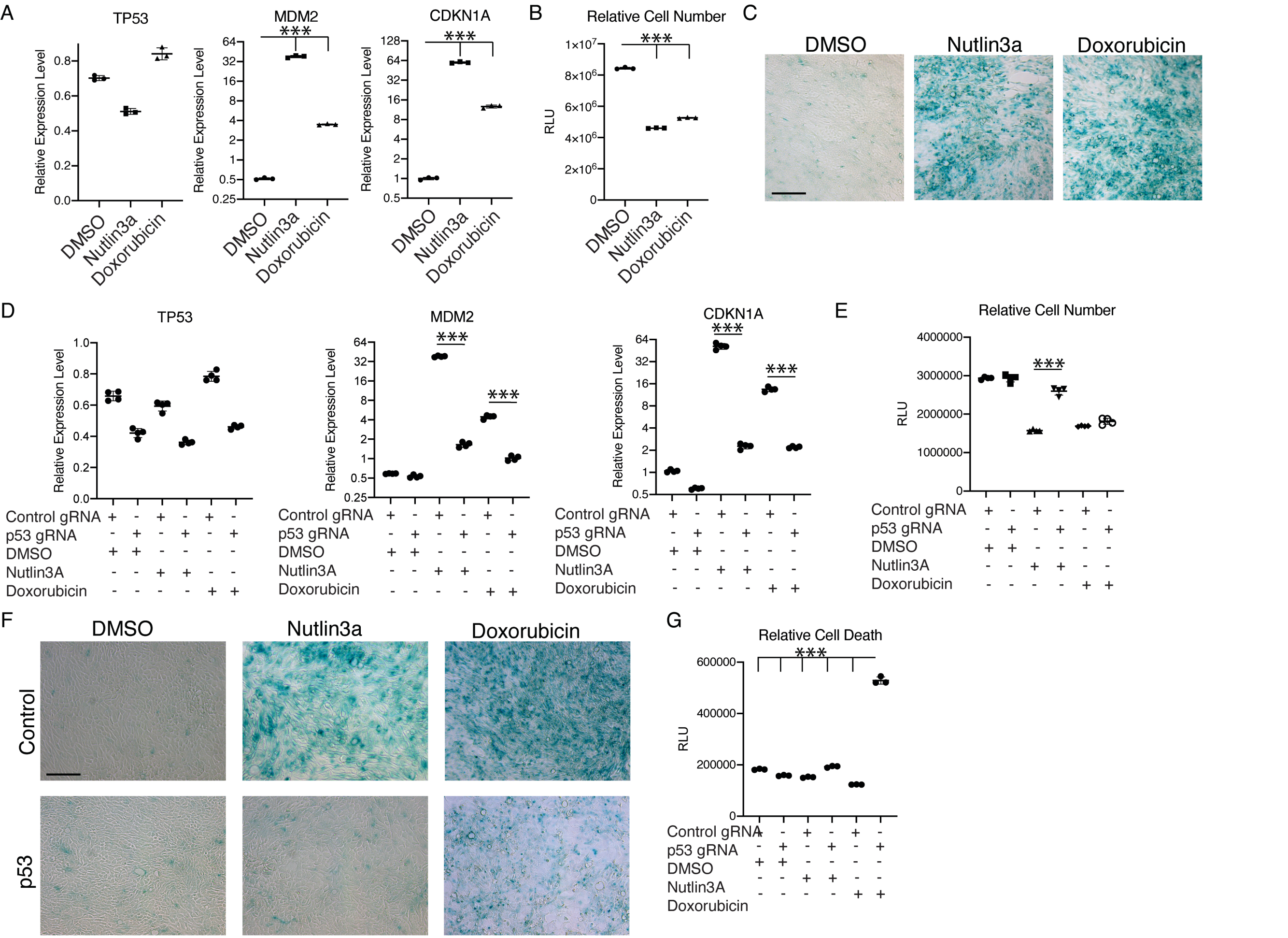

Supplementary Figure 7. p53 is sufficient and necessary for induction of cellular senescence. (A) Gene expression in NHBE cultures at 24hrs post treatment (mean ± SD), n=3. ***P < 0.005 and FC>2 (Tukey's multiple comparisons test). (B) Relative cell number, via CellTiter-glo, of NHBE cultures at day 3 of treatment (mean ± SD, n=3. ***P < 0.005 (Tukey's multiple comparisons test). (C) SA-βGAL staining of NHBE cultures at day 5 of treatment. (D) Gene expression in Control or p53 CRISPR targeted NHBE cultures at 24hrs post treatment (mean ± SD, n=3. ***P < 0.005 and FC>2 (unpaired 2-tailed Student's t tests).(E) Relative cell number of CRISPR targeted NHBE cultures at day 3 of treatment (mean ± SD, n=3. ***P < 0.005 (unpaired 2-tailed Student's t tests). (F) SA-βGAL staining of CRISPR targeted NHBE cultures at day 5 of treatment. (G) Relative cell death, via CytoTox-Glo, in CRISPR targeted NHBE cultures at day 5 (mean ± SD, n=3. ***P < 0.005 (Tukey's multiple comparisons test). Scale Bars: 100μm in C, F. RLU: Relative light units.

Supplemental Table 1.

Consensus Lung Epithelial Senescence Signature

ABCA1	CLDN4	FANK1	IL1RN	MYLK	REEP	SP6	TRAF3IP3
ACKR3	COL5A1	FAT2	IL32	NAGK	RHBDL2	SPRR1A	TREM2
ACSF2	CPA4	FBN1	IL33	NCCRP1	RHCG	SPRR1B	TRIM16
ADAMTS7	CPPED1	FER1L4	IL36G	NDST1	RHOV	SPRR2A	TUBA1A
ADAMTSL4	CRABP2	FGF11	IRF6	NRCAM	RNF212B	SRPX2	TUBB2A
AIM1L	CRYAB	FHDC1	IVL	NTN1	RRAD	SSC4D	UCP2
ALOX15B	CST6	FLG	KIAA0922	NYNRIN	S100A4	STARD5	ULBP2
ANGPTL4	CTNNBIP1	FLG-AS1	KLRC4	OTUB2	S100A8	STC1	UPK3B
ANKRD22	CUX2	FRMD8	KLRK1	OVOL1	S100A9	SUGCT	VGLL3
ANXA4	CXCL1	GALNT5	KRT13	PAM	SAA1	SYNPO	WDR63
ARHGEF4	CXCL17	GAS6-AS1	KRT16	PAMR1	SCD5	SYTL4	XG
ATL1	CYB5R1	GBP3	KRT23	PAPLN	SCN4B	TACSTD2	ZNF185
B4GALNT4	DAPK1	GCNT4	KRT6A	PAQR7	SEC14L2	TAGLN	ZNF429
C1orf74	DHRS1	GGT1	KRT6B	PCBP4	SEPT5-GP1BB	TCEA2	ZNF488
C1QTNF1	DHRS12	GJB4	LAYN	PDE9A	SERPINB13	TENM2	ZNF702P
C1QTNF6	DIXDC1	GPX3	LDLRAD2	PDLIM1	SERPINB3	THSD4	ZNF704
C3	DKK3	GRHL3	LOC100862671	PGBD5	SERPINB7	TLL2	ZNF750
CALML3	DOCK8	HEG1	LOC101928100	PIK3IP1	SERPINB9	TMBIM1	
CALML3-AS1	DPYSL4	HEPHL1	LOC101928281	PLA2G4F	SH3BGRL	TMEM132A	
CAPN3	DTNA	HES2	LOC101930241	PLK2	SH3TC2	TMEM217	
CCND1	DUOXA1	HIST1H1C	LOC103021296	PLOD2	SLC15A3	TMEM229B	
CCND2	ECM1	HIST1H2AC	LRP1	PORCN	SLC16A2	TMEM40	
CCRN4L	EDN1	HIST1H2BD	LY6D	PPP2R2C	SLC1A1	TMEM63C	
CD24	EHD3	HIST1H2BK	LYPD3	PROM2	SLC31A2	TMEM79	
CD82	ELFN2	HIST2H2BE	MAGED1	PRR5L	SLC39A11	TMPRSS13	
CEACAM6	ENC1	HSPG2	MATN2	PRSS22	SLC44A2	TMPRSS4	
CHST2	ENTPD3	ID3	MFI2	PRSS23	SLC46A3	TNFSF10	
CKB	EPHA4	IGFBP2	MMP28	PTGES	SLC6A11	TOM1L2	
CLCA2	EPHB3	IKZF2	MXRA5	RAB7B	SLC9A3	TP53I3	
CLDN1	ESPN	IL12RB2	MYH14	REC	SNCG	TPM1	

Supplemental Table 2.

Consensus Fibroblast Senescence Signature

ABCA3	CYB5D2	LYNX1	RRM2B
ABCA7	DHRS3	MANSC1	SCG50
ABCA8	DPP4	MGARP	SECTM1
AC007388.1	DRAM1	MGP	SERPING1
ACSS3	DYNC2LI1	MGST2	SLC1A1
ADAMTS5	FAM162A	MORN4	SLC40A1
ADGRL1	FAM198B	MXI1	SOD2
AMPD3	FAM43A	NALCN	SPATA18
AMZ2P1	FAXDC2	NCOA7	ST3GAL5
ANK2	FBXO32	NEAT1	SULF1
AP001972.5	FER1L4	NTN4	SULF2
ARHGEF37	FIBIN	ORAI3	SVEP1
ARRB1	GFRA1	PAPPA	TCEA2
BAMBI	GPR155	PCMTD1	TLCD2
BMP4	GPRC5B	PCSK5	TM7SF2
BTG2	HIST1H1C	PDGFD	TMEM170B
C1QTNF1	HIST1H2AC	PIK3IP1	TMEM176B
C1S	HIST1H2BD	PLD1	TMEM178B
C3	HIST1H3E	PLXNC1	TP53INP1
CAMK1D	HIST1H4H	PODN	TXNIP
CBLN3	HIST1H4I	PPP1R3C	VSIR
CCND2	HNMT	PRRG1	WDR63
CCPG1	IFI6	PTCHD4	WFDC21P
COL4A5	IGDCC4	QPRT	YPEL2
CPE	JAM2	RARRES2	ZMAT3
CPZ	JMY	RDH10	ZNF219
CREBRF	KCNJ2	RGCC	ZNF667
CRELD1	KLHL24	RHBDL1	
CTSF	LACC1	RILP	
CTSO	LRRK2	RPS6KA2	

Supplemental Table 3.

Overlap between consensus epithelial and fibroblast senescence signatures

C1QTNF1
C3
CCND2
FER1L4
HIST1H1C
HIST1H2AC
HIST1H2BD
PIK3IP1
SLC1A1
TCEA2
WDR63

Supplemental Table 4.

Demographic information for human explant samples

Sample	Diagnosis	Gender	Age	Ethnicity	Smoking History
N1	Control	Male	68	Hispanic/Latino	2 packs/wk for 20 years
N2	Control	Female	70	Hispanic/Latino	No
N3	Control	Female	59	Caucasian	No
N4	Control	Male	67	Caucasian	No
I1	IPF	Female	70	Hispanic/Latino	No
I2	IPF	Female	70	Caucasian	Former
I3	IPF	Male	73	Caucasian	Former
I4	IPF	Male	62	Caucasian	Former
S1	SSc-ILD	Female	53	Hispanic/Latino	Former
S2	SSc-ILD	Male	52	South Asian	No
S3	SSc-ILD	Female	56	African American	No

Supplemental Table 6.

Taqman assays utilized in qPCR studies

Gene	Assay
BUB1	Hs01557695_m1
MKI67	Hs04260396_g1
PCNA	Hs00427214_g1
E2F1	Hs00153451_m1
DSC3	Hs00170032_m1
DSG3	Hs00951897_m1
FLG	Hs00856927_g1
IVL	Hs00846307_s1
KRT1	Hs00196158_m1
KRT10	Hs00166289_m1
KRT14	Hs00265033_m1
LOR	Hs01894962_s1
TGM1	Hs00165929_m1
TP63	Hs00978340_m1
EPCAM	Hs00901885_m1
CDKN2A	Hs00923894_m1
CALML3	Hs00359114_s1
CLCA2	Hs00998924_m1
FOLR3	Hs01549264_m1
LCN2	Hs01008571_m1
LY6D	Hs01008571_m1
RHOV	Hs00370444_g1
SPRR1B	Hs00824893_m1
TSPAN1	Hs00371661_m1
CCND1	Hs00765553_m1
ANGPTL4	Hs01101123_g1
IL1B	Hs01555410_m1
IL6	Hs00174131_m1
CXCL8	Hs00174103_m1
IL32	Hs00992441_m1
IL33	Hs04931857_m1
CXCL6	Hs00605742_g1
